# Supplementary material for: Overcoming adaptive resistance to anti-VEGF therapy by targeting CD5L
Source: Nat Commun. 2023 Apr 26;14:2407. doi: 10.1038/s41467-023-36910-5 (PMC10133315; doi:10.1038/s41467-023-36910-5)
Supplement: Supplementary file 1 — Supplementary Information [file 41467_2023_36910_MOESM1_ESM.pdf]

SUPPLEMENTAL FIGURE LEGENDS

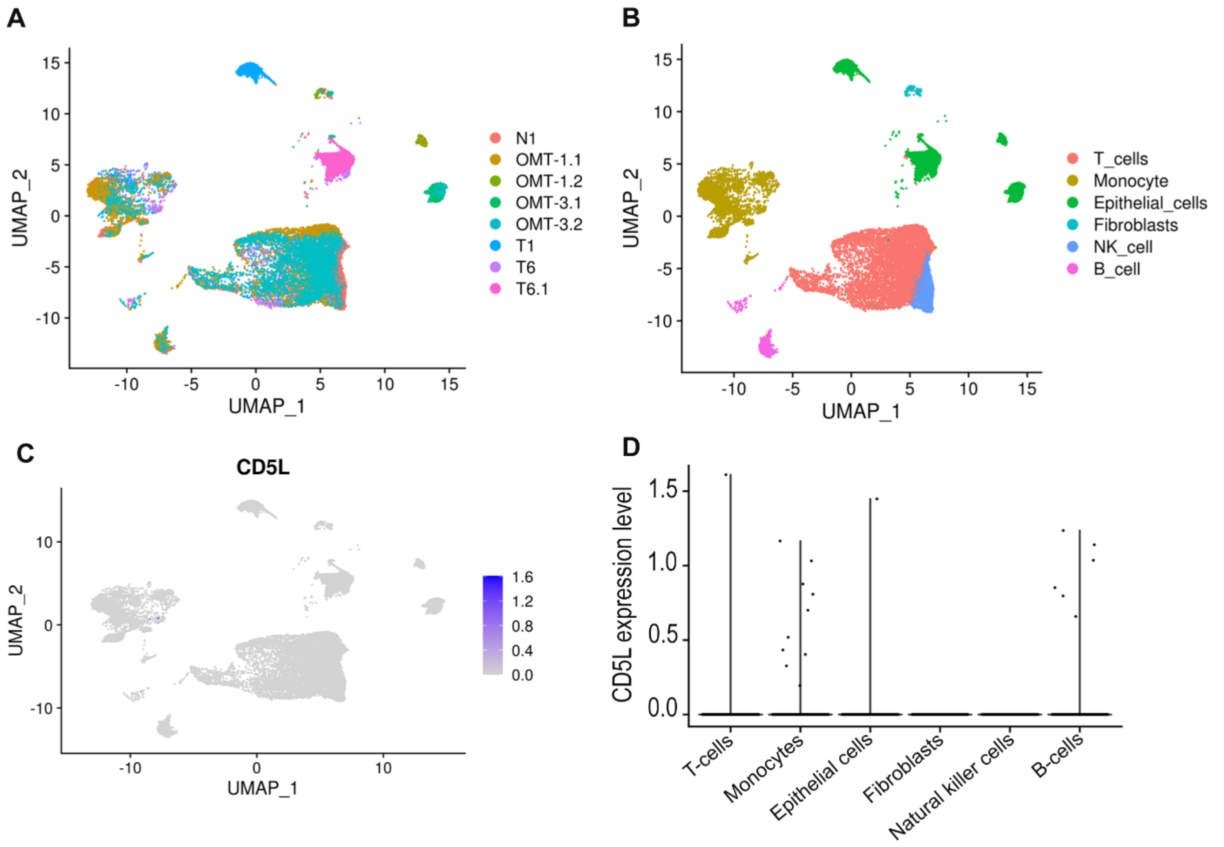

Fig. S1. *CD5L* expression in human ovarian tumors. A total of 5 freshly resected high-grade serous ovarian cancer (HGSC) specimens were collected. Samples were processed using 10X genomic Chromium Single cell 3' v3. UMAP visualization of major cell types was used for subsequent analysis by **(A)** sample and **(B)** cell type. **(C)** UMAP visualization of *CD5L* expression. **(D)** Violin plot of *CD5L* expression in the major cell types. Samples with similar nomenclature such as OMT-1.1 and OMT-1.2 refers to the same patient which were sorted with CD45+ and CD45- before the single cell analysis. The single cell analysis data was deposited in GEO (Accession number GSE181955).

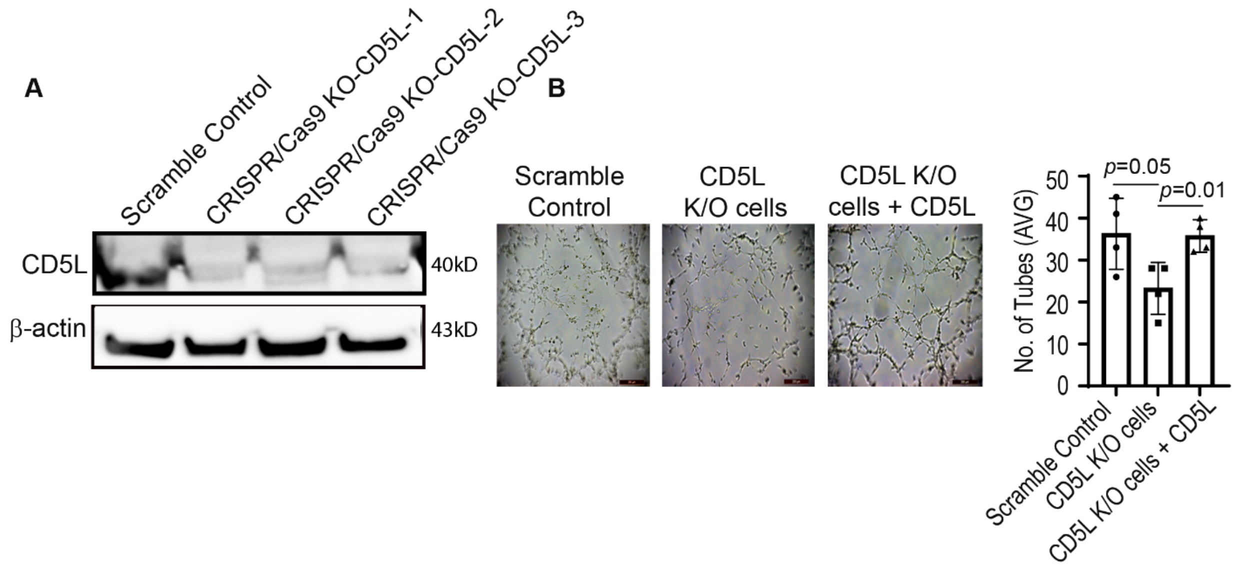

Fig. S2. Effect of CD5L silencing on angiogenesis. (A) CD5L protein expression in *CD5L KO* RF24 cells and **(B)** tube formation of *CD5L KO* RF24 cells treated with CD5L recombinant protein. Data represented as mean values ± SD, determined by two-tailed Student 's *t* test (n = 4 biological independent experiments; scale bar = 200 μm).

CD5L promoter sequence

```
  1 ATAACAAATC TGTATATTGG ACCCTCTGCT TAGCAGTGAG AAAGCAGGTT TGAAGACAAT
 61 AAAGCCAGGC CTGTATGTGG AGATTGGCAC CATGACCCTG GATTATGTTT TATGGGATAT
121 GCCTCCAAGG GCAACTCTGC ATTTATCCCA TGGCCTCTGG CTTCCAAATT TTCTAGGCAC
181 TTCCCTCTGG GGTAAAGGAA TCAAAATTGG CTTATTTCCC TTGCAAACCA TCCTTGGCGA
241 CTAAGTACTG CCTTGTCTTC TACTGAGAGA TGGCCATATT TTTGGCACCT GCCTTTCCTT
301 GTGGGAGGTG TTTCCTCTGT TTACATAGCA AAGGGGCTGT AGGAGAGACA GACGGAGCTG
361 GACTTACAGC AGAACCAAGT CATGATAGCC TGTTTCTATT TTGTTTTCAG CATTTTTCCG
421 CCAGTTCTGG CCACCTCCTT TCCTCTGGAA CATGCTGATT TCAGCAAGTC CAGCTCTGTC
481 AGCTCTGCCC CCCGAGTCTA TTGGTTTCTG ATCATCTGAT AATGCTTTGC CTGCACTCAG
541 GACCTGTCTT TGTCCCTCCT CTTAACATAC TTGCAGCTAA AACTAAATAT TGCTGCTTGG
601 GGACCTCCTT CTAGCCTTAA ATTTCAGCTC ATCACCTTCA CCTGCCTTGG TCATGGCTCT
661 GCTATTCTCC TTGATCCTTG GTGAGTATCT CTGCACCTGT TGGTTTAGGC TTCAGAGTTT
721 TCTGGCACTT TGATTAGGAG AACTTTCTCC CCGC
```

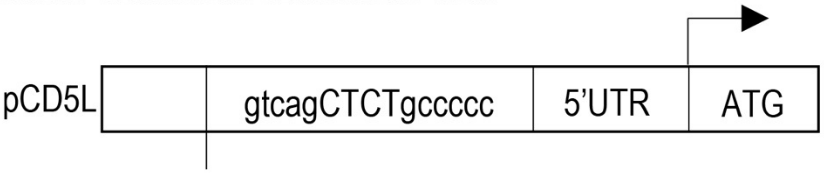

Fig. S3. Promoter sequence of CD5L. PPARG binding site identified in red. Lower image represents *CD5L* promoter construct with critical base pairs in PPARG binding highlighted (CTCT).

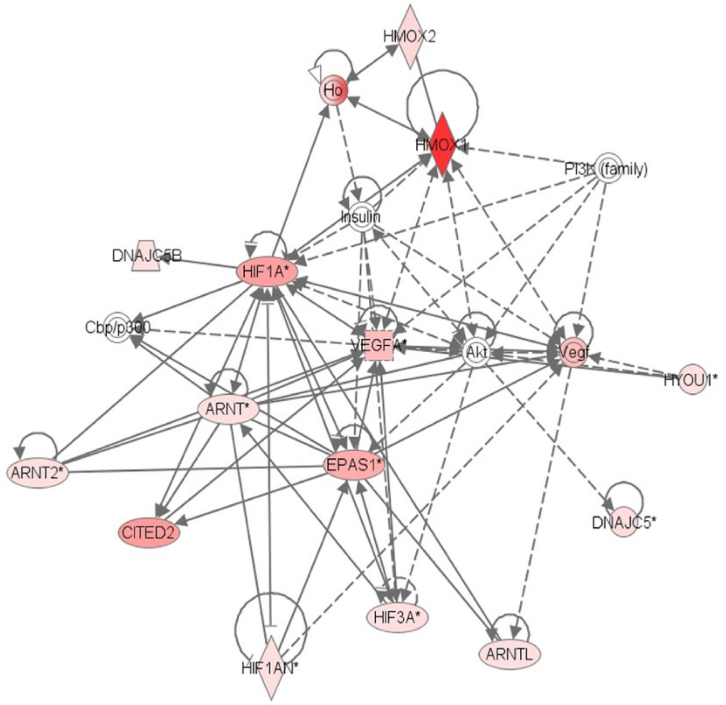

Fig. S4. Ingenuity pathway analysis (IPA) of anti-VEGF antibody (AVA) resistant mouse tumor endothelial cells. Resistance to anti-VEGF therapy is associated with increased hypoxia signaling. IPA analysis performed on gene expression profile presented in Figure 1.

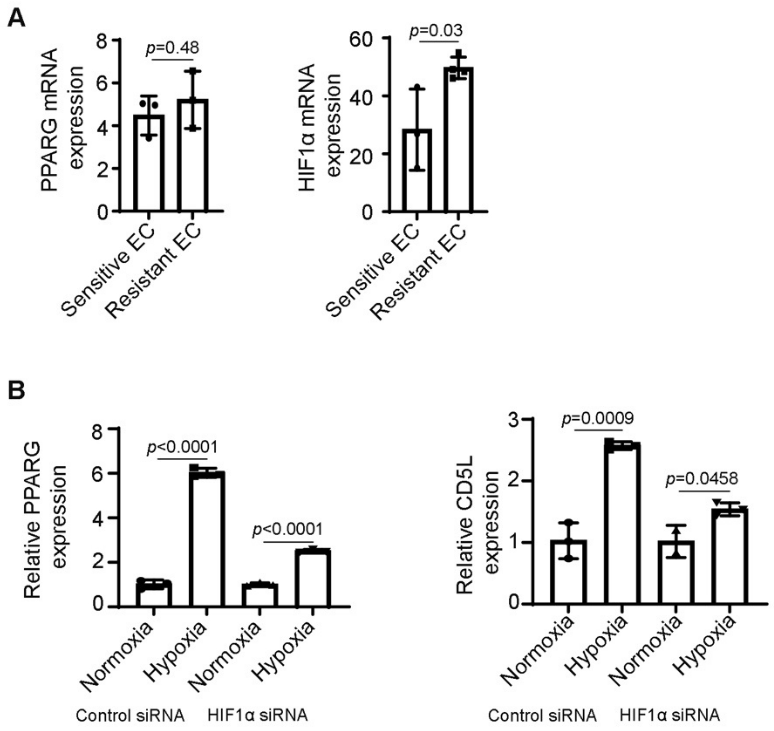

Fig. S5. Upregulation of PPARG and HIF1α in anti-VEGF antibody-resistant tumor endothelial cells under hypoxic condition. (A) *PPARG* and *HIF1α* expression in B20 sensitive and resistant endothelial cells isolated from SKOV3ip1 ovarian tumor (related to microarray data, Fig. 1A). **(B)** *PPARG* and *CD5L* expression in RF24 cells treated with *HIF1α* siRNA versus control siRNA in normoxic and hypoxic conditions. Data represented as mean values ± SD, determined by two-tailed Student ‘s *t* test (n = 2-3 biologically independent experiments).

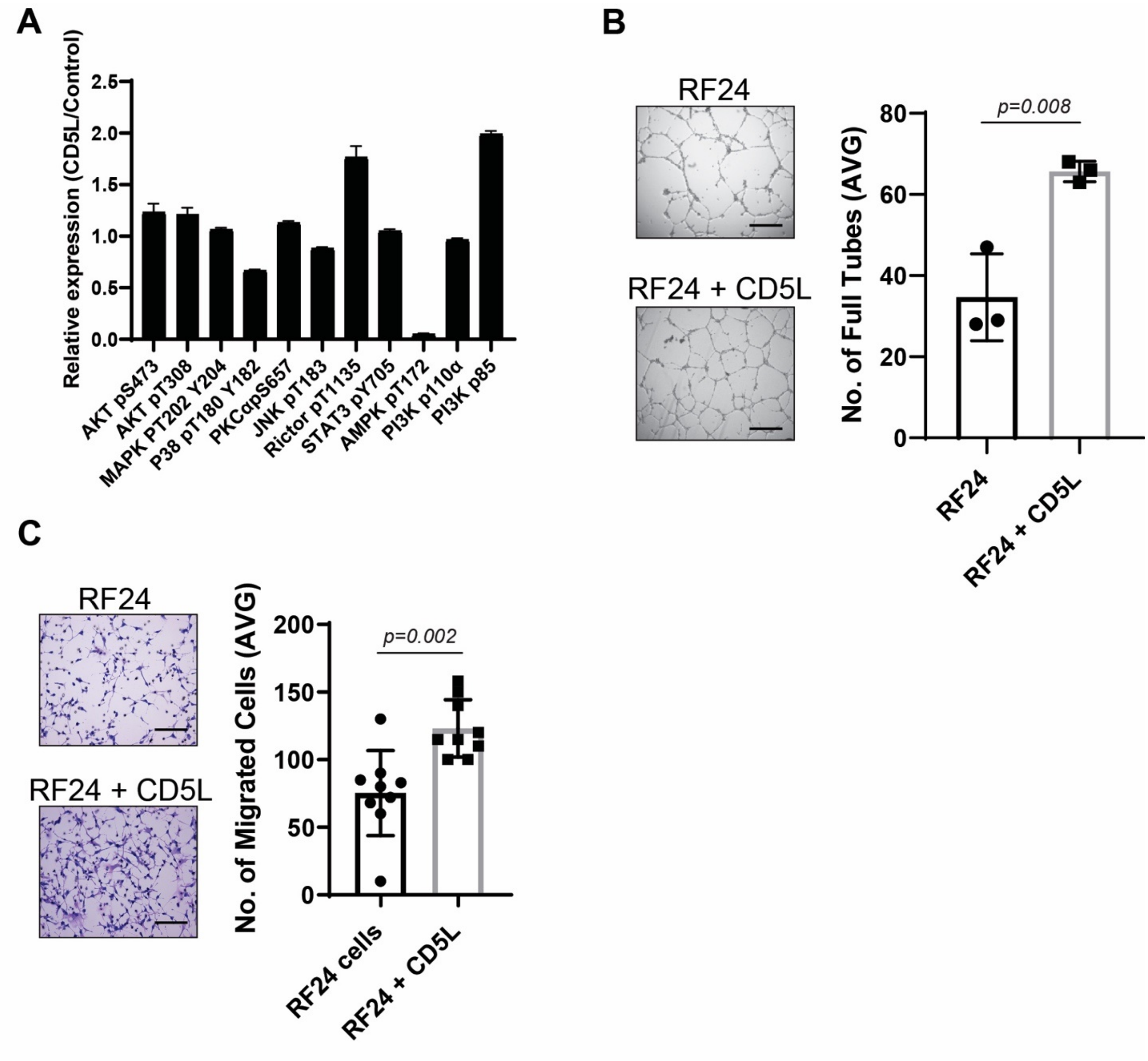

Fig. S6. Effect of exogenous CD5L protein treatment on RF24 endothelial cells. (A) Reverse phase protein array (RPPA) analysis of RF24 endothelial cells treated with CD5L protein *versus* control. **(B, C)** Tube formation (B) and cell migration (C) of RF24 cells alone or after addition of 400 ng/ml CD5L protein. Data represented as mean values ± SD, determined by two-tailed Student 's *t* test (n = 3 biologically independent experiments; scale bar = 500 μm for B and 200 μm for C).

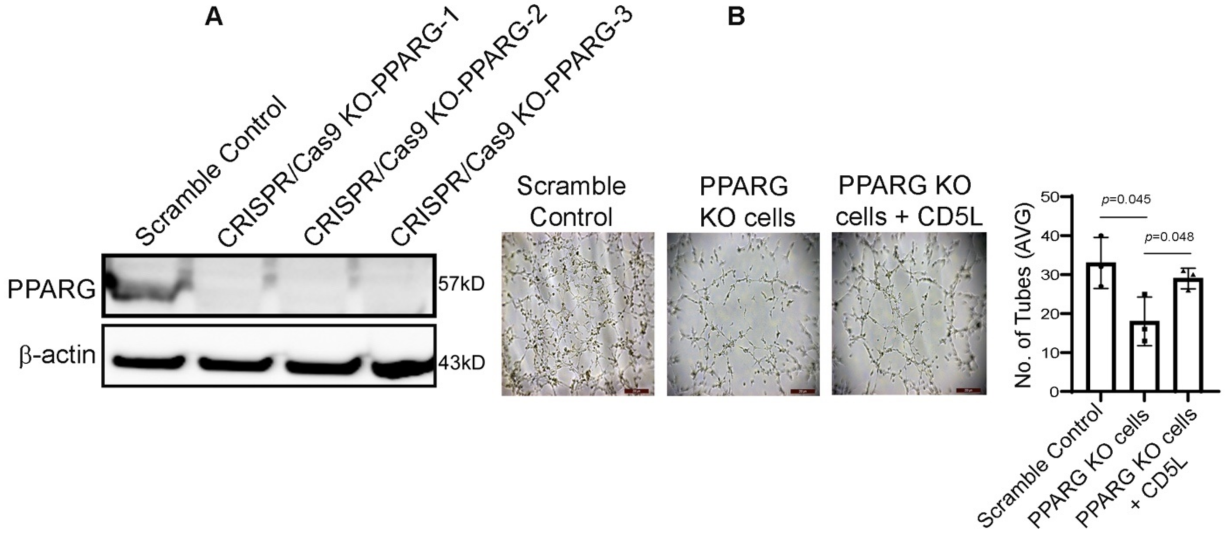

Fig. S7. Effect of PPARG silencing on angiogenesis. (A) PPARG protein expression in *PPARG* KO RF24 cells and **(B)** tube formation of *PPARG* KO RF24 cells treated with CD5L recombinant protein. Data represented as mean values ± SD, determined by two-tailed Student 's *t* test (n = 3 biologically independent experiments; scale bar = 200 μm).

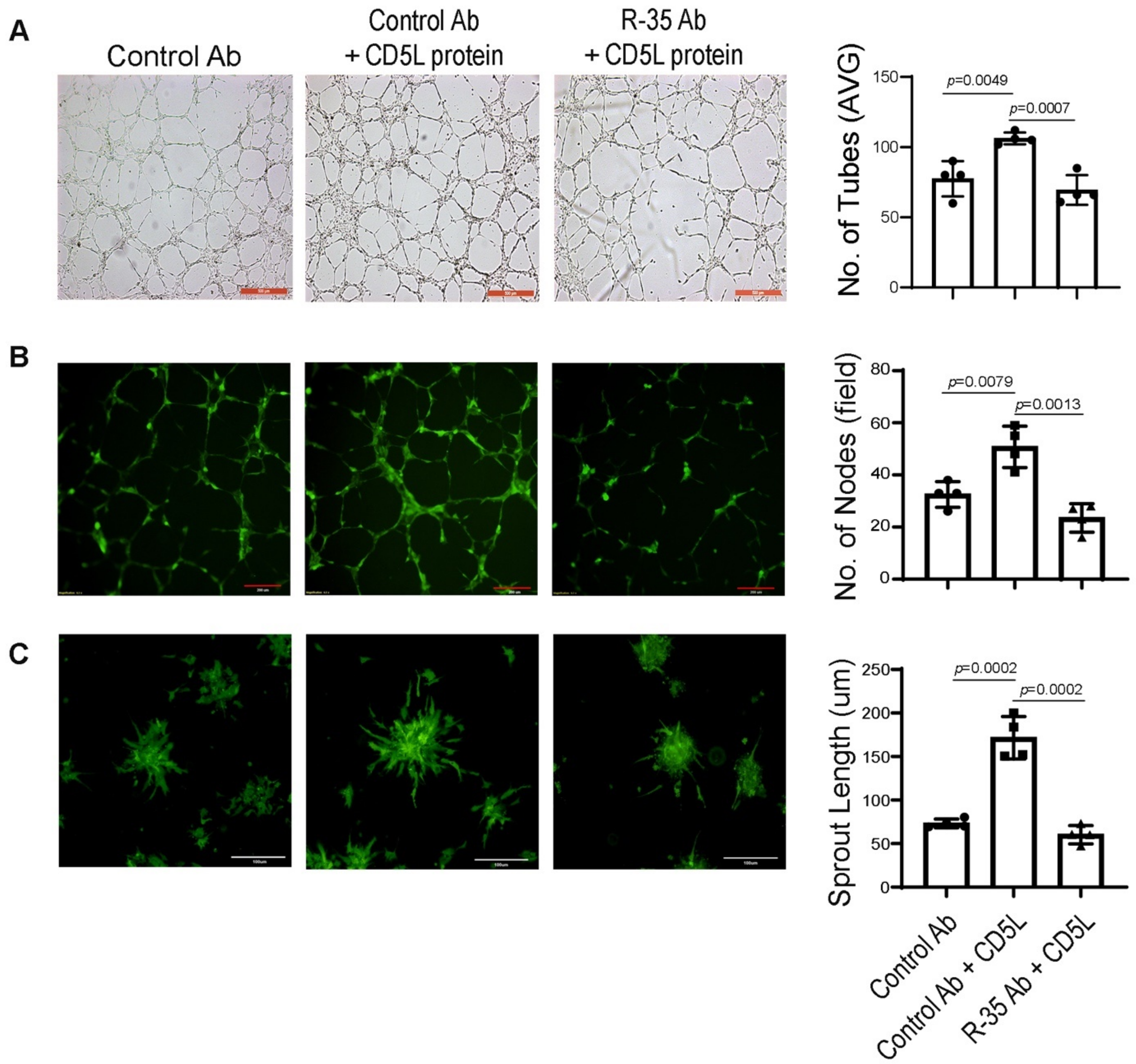

Fig. S8. Effect of CD5L on tube formation and capillary formation. Tube formation of **(A)** human pulmonary artery endothelial cells (HPAECs; scale bar = 500 μm) and **(B)** human umbilical venous endothelial cells (GFP-HUVECs) treated with CD5L recombinant protein and R-35 antibody; scale bar = 200 μm. **(C)** Capillary formation of GFP-HUVECs treated with CD5L recombinant protein and R-35 antibody (scale bar = 100 μm). Data represented as mean values ± SD, determined by two-tailed Student 's *t* test (n = 4 biologically independent experiments).

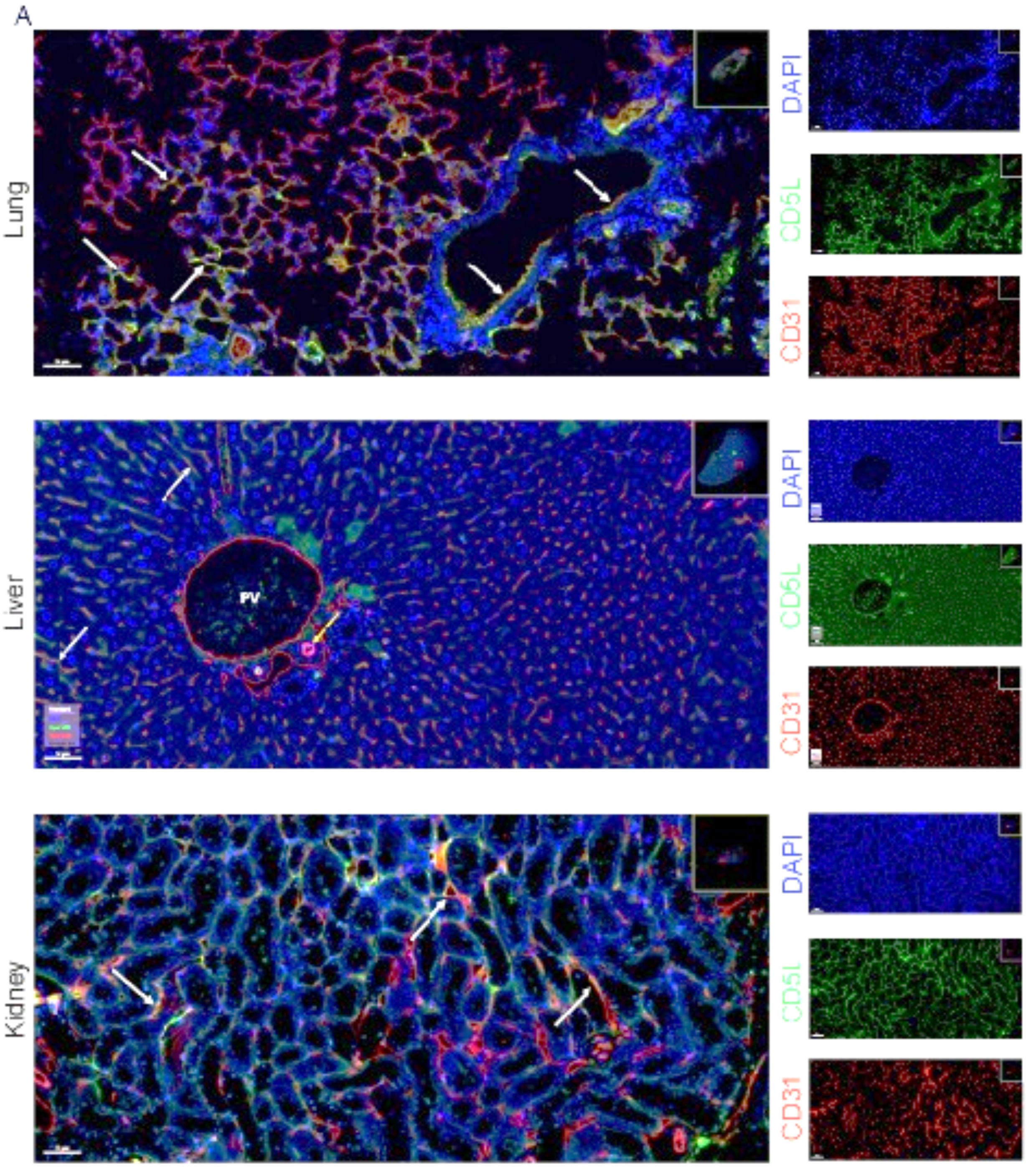

A
Lung
Liver
Kidney
PV
DAPI
CD5L
CD31
DAPI
CD5L
CD31
DAPI
CD5L
CD31

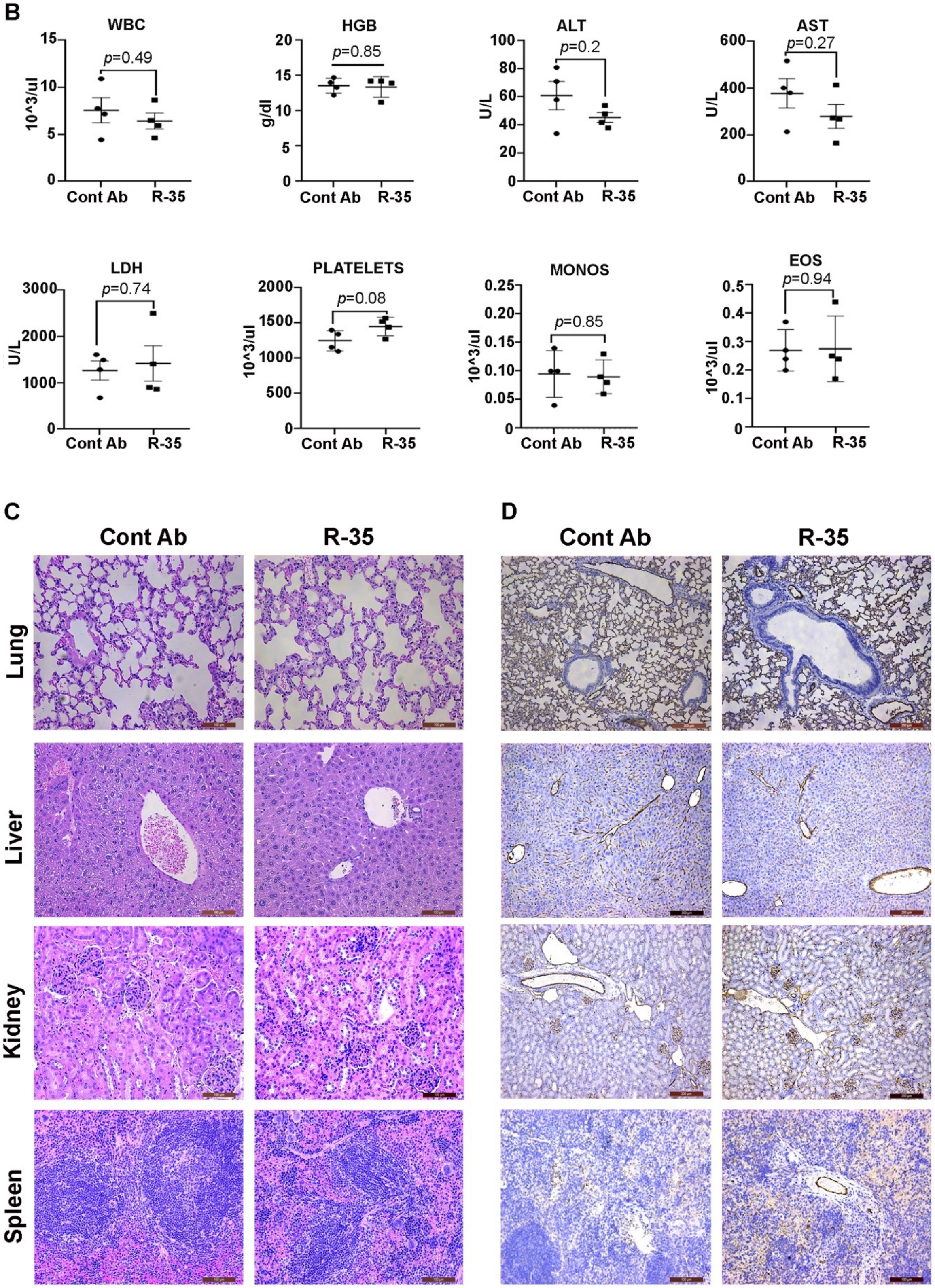
B
WBC
10^3/ul
p=0.49
Cont Ab
R-35
HGB
g/dl
p=0.85
ALT
U/L
p=0.2
AST
U/L
p=0.27
LDH
U/L
p=0.74
PLATELETS
10^3/ul
p=0.08
MONOS
10^3/ul
p=0.85
EOS
10^3/ul
p=0.94
C
D
Cont Ab
R-35
Lung
Liver
Kidney
Spleen

Fig. S9. CD5L expression in lung, liver and kidney endothelial cells and effect of CD5L blocking antibody R-35 on normal mice. (A) Expression of CD5L in endothelial cells of normal organs from C57BL/6 mice (n = 4 mice) - continuous endothelium of lung (white arrows; scale bar = 50 µm); hepatic arterial (denoted by yellow arrow), portal venous (PV), and discontinuous (white arrows; scale bar = 50 µm) endothelium in liver; capillary epithelium of kidney (white arrows and scale bar = 50 µm). **(B)** Serum levels of white blood cells (WBC), hemoglobin (HGB), aspartate aminotransferase (AST), alanine aminotransferase (ALT), lactate dehydrogenase (LDH), platelets, monocytes (MONOS), and eosinophils (EOS) in normal C57BL/6 mice treated with either control antibody or R-35 antibody. **(C)** Hematoxylin and eosin (H&E) staining of lung, liver, kidney, and spleen of normal C57BL/6 mice treated with either control antibody or R-35 antibody. **(D)** CD31 staining of endothelial cells of normal C57BL/6 mice treated with either control antibody or R-35 antibody. Data represented as mean values ± SEM, determined by two-tailed Student 's *t* test (n = 4 biologically independent experiments for B, C and D. Scale bar = 100 µm for both C and D).

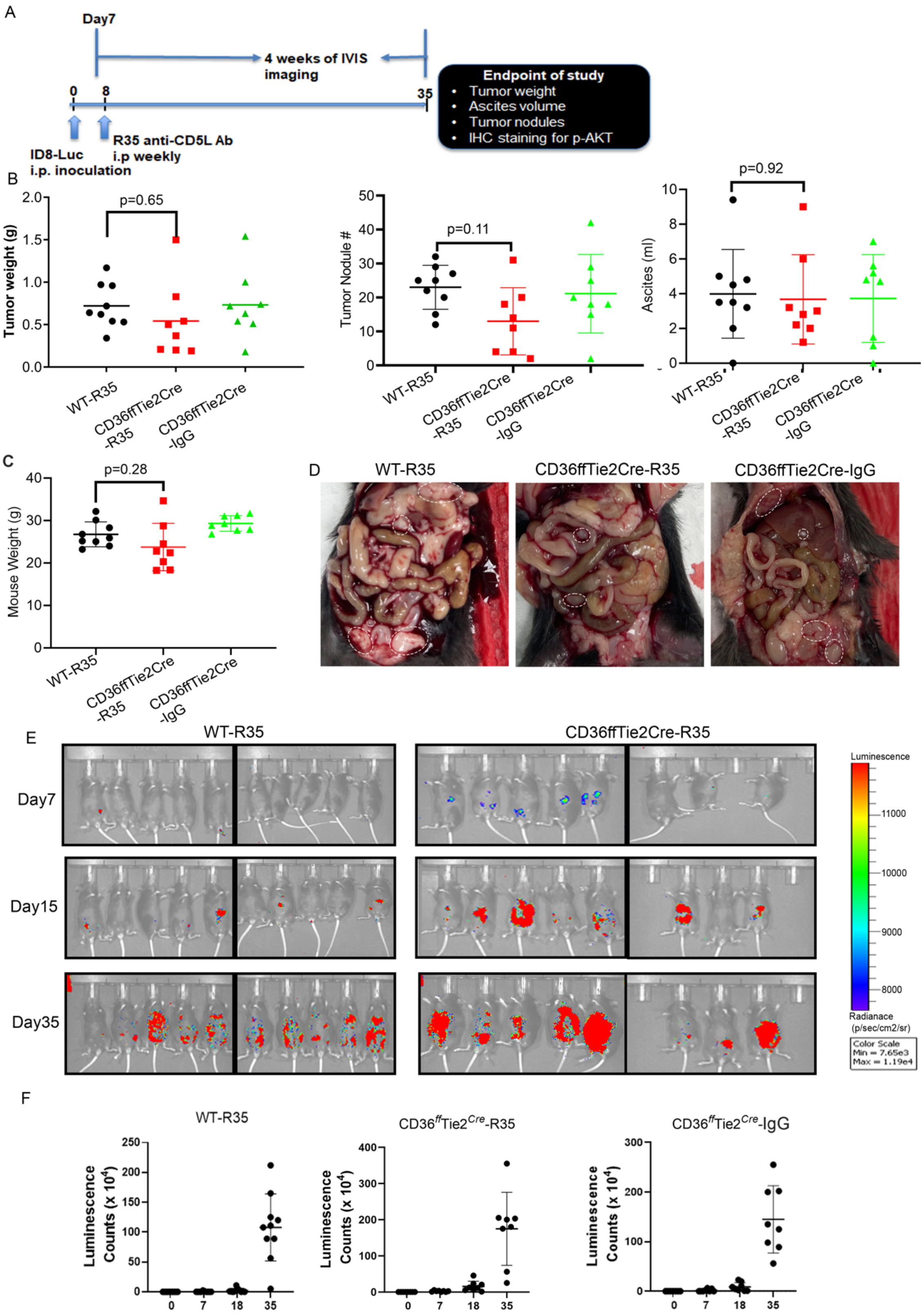
A
Day7
4 weeks of IVIS imaging
0
8
35
Endpoint of study
Tumor weight
Ascites volume
Tumor nodules
IHC staining for p-AKT
ID8-Luc i.p. inoculation
R35 anti-CD5L Ab i.p weekly
B
Tumor weight (g)
p=0.65
Tumor Nodule #
p=0.11
Ascites (ml)
p=0.92
WT-R35
CD36ffTie2Cre -R35
CD36ffTie2Cre -IgG
C
Mouse Weight (g)
p=0.28
D
WT-R35
CD36ffTie2Cre-R35
CD36ffTie2Cre-IgG
E
WT-R35
CD36ffTie2Cre-R35
Day7
Day15
Day35
Luminescence
Radianace (p/sec/cm2/sr)
Color Scale Min = 7.65e3 Max = 1.19e4
F
WT-R35
CD36ffTie2Cre-R35
CD36ffTie2Cre-IgG
Luminescence Counts (x 10^4)

Fig. S10. ***In vivo*** **study of anti-CD5L-antibody (R-35) therapeutic effect on syngeneic ovarian cancer models using endothelial-specific *CD36* knockout (CD36$^{\mathbf{flox/flox}}$ Tie2$^{\mathbf{cre}}$) strain. (A)** Mice from the CD36$^{flox/flox}$ Tie2^{cre} model were treated with anti-CD5L antibody (R-35) after inoculation with ID8 cells labeled with luciferase. **(B)** Tumor weight, number of tumor nodules, and volume of ascites fluid. **(C)** Body weight of each mouse recorded at the time of necropsy. Data represented as mean values ± SD determined by two-tailed nonparametric Student's *t* test (n = 9 for WT-R35; n = 8 for CD36$^{flox/flox}$Tie2^{cre}-R35 and CD36$^{flox/flox}$Tie2^{cre}-IgG groups, respectively). **(D)** Representative gross images from C57BL/6 , CD36$^{flox/flox}$Tie2^{Cre} mice that received R-35 or control IgG antibody treatment. **(E)** Gross images for bioluminescence in C57BL/6 or CD36$^{flox/flox}$Tie2^{Cre} mice. **(F)** Quantification of bioluminescence imaging for the three groups of mice.

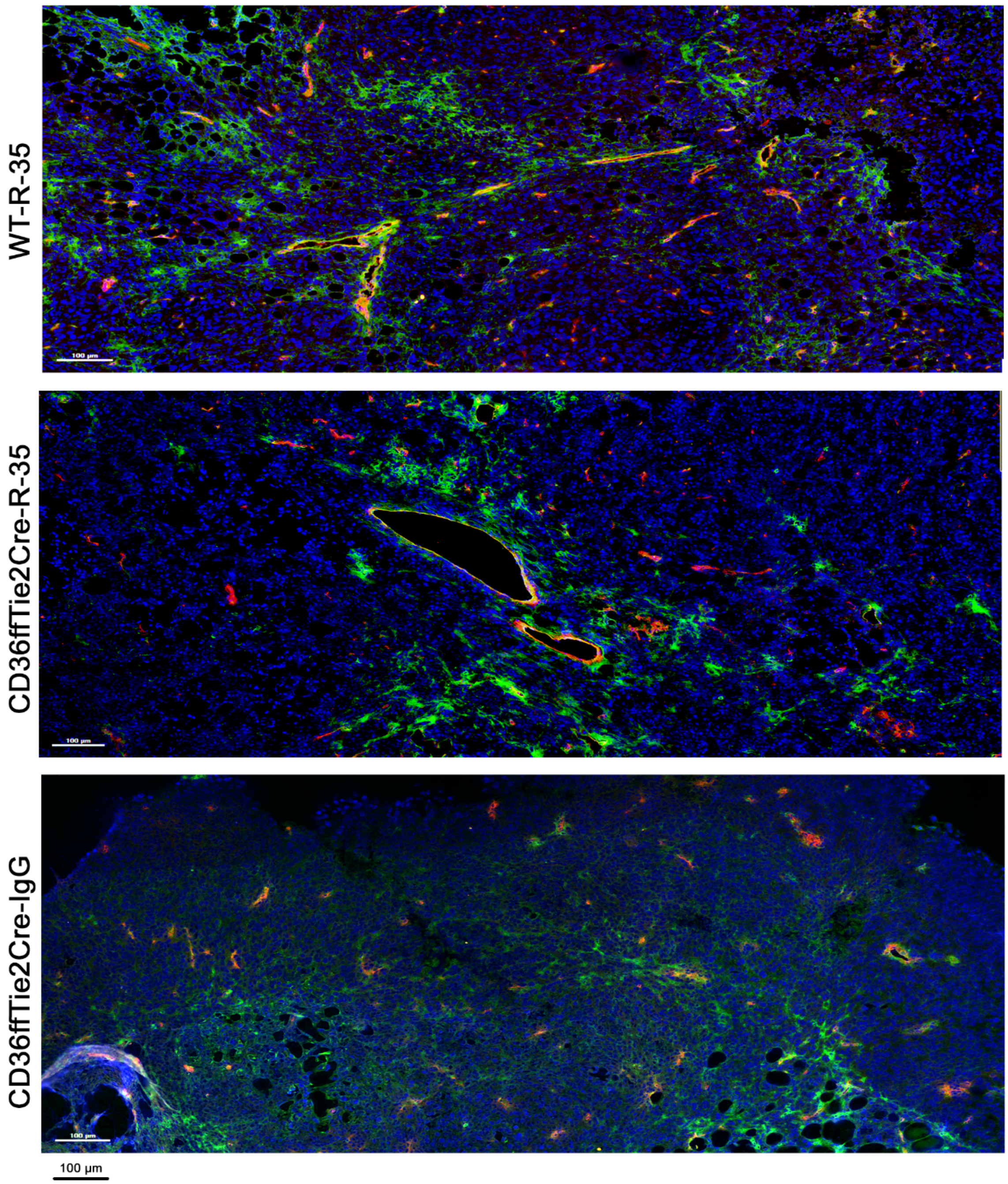

Fig. S11. Effects of anti-CD5L antibody (R-35) on angiogenesis in endothelial-specific *CD36* knockout ID8 tumors. CD31(Red) and pAKT (Green) immunofluorescence staining of tumors from CD36 endothelial-specific KO mice treated with R-35 antibody or IgG (n = 4 biologically independent experiments; scale bar = 100 µm).

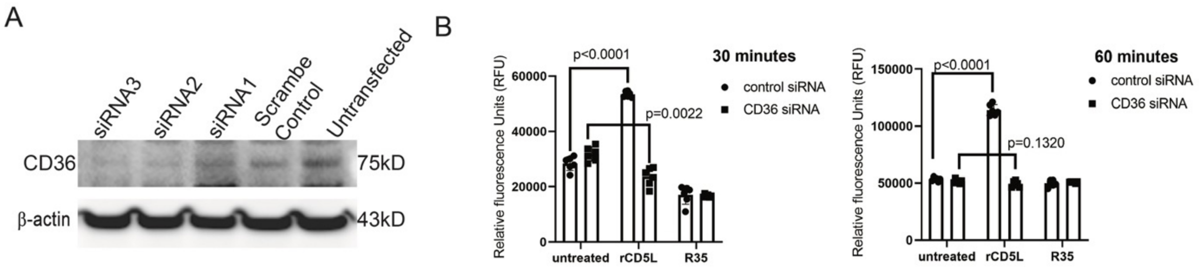

Fig. S12. Fatty acid uptake in RF24 cells with CD5L stimulation or CD36 knockdown. (A) CD36 protein expression in RF24 cells treated with human CD36 siRNA *versus* control siRNA. **(B)** Relative exogenous fatty acid uptake in RF24 cells treated with control, rCD5L, anti-CD5L antibody (R-35) after serum starvation for 30 min and 60 min and supplementation with fluorescently labeled dodecanoic acid. Data represented as mean values ± SD determined by two-tailed Student ‘s *t* test (n = 6 biological independent experiments).

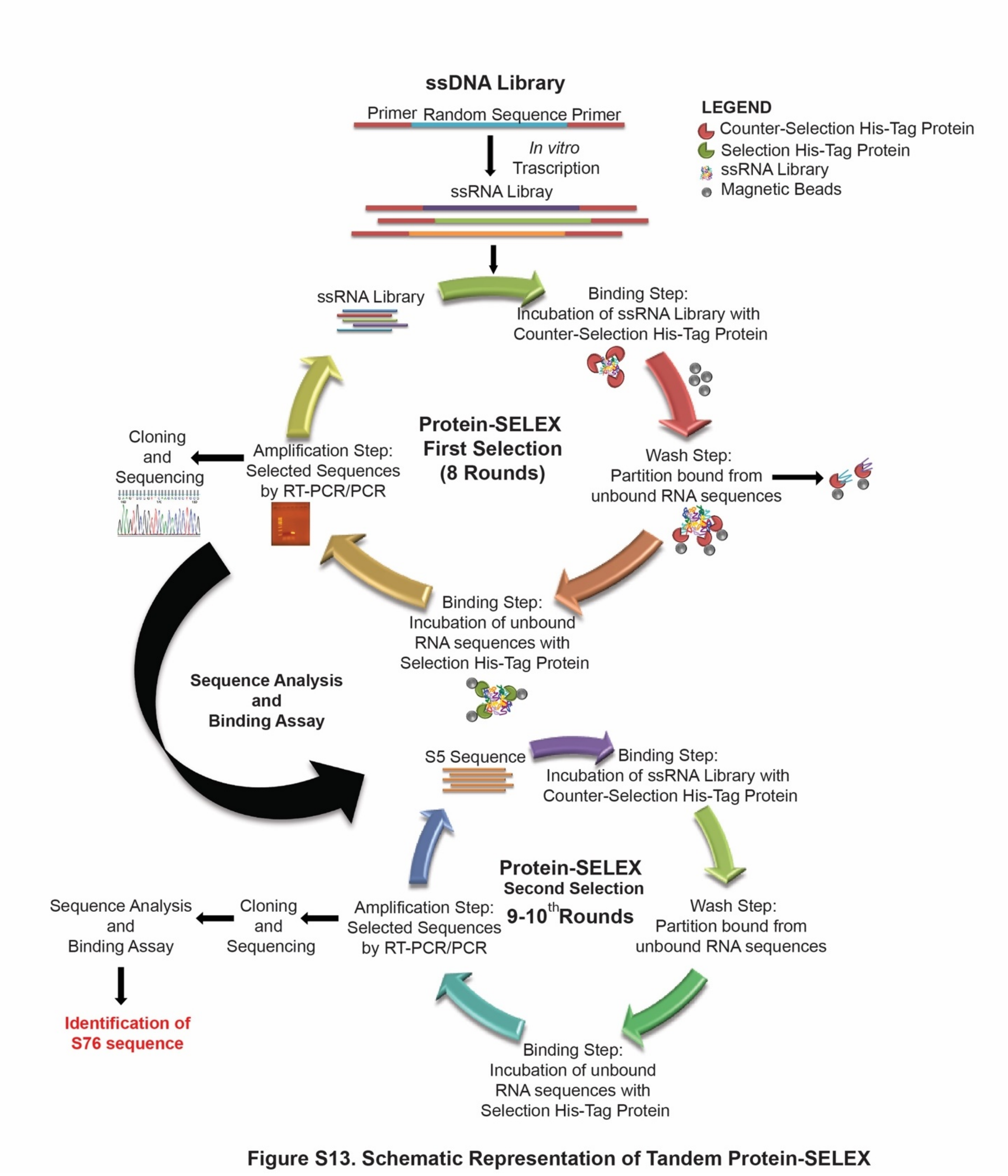

Fig. S13. Schematic Representation of Tandem Protein-SELEX. S76.T selection by the tandem protein-SELEX method.

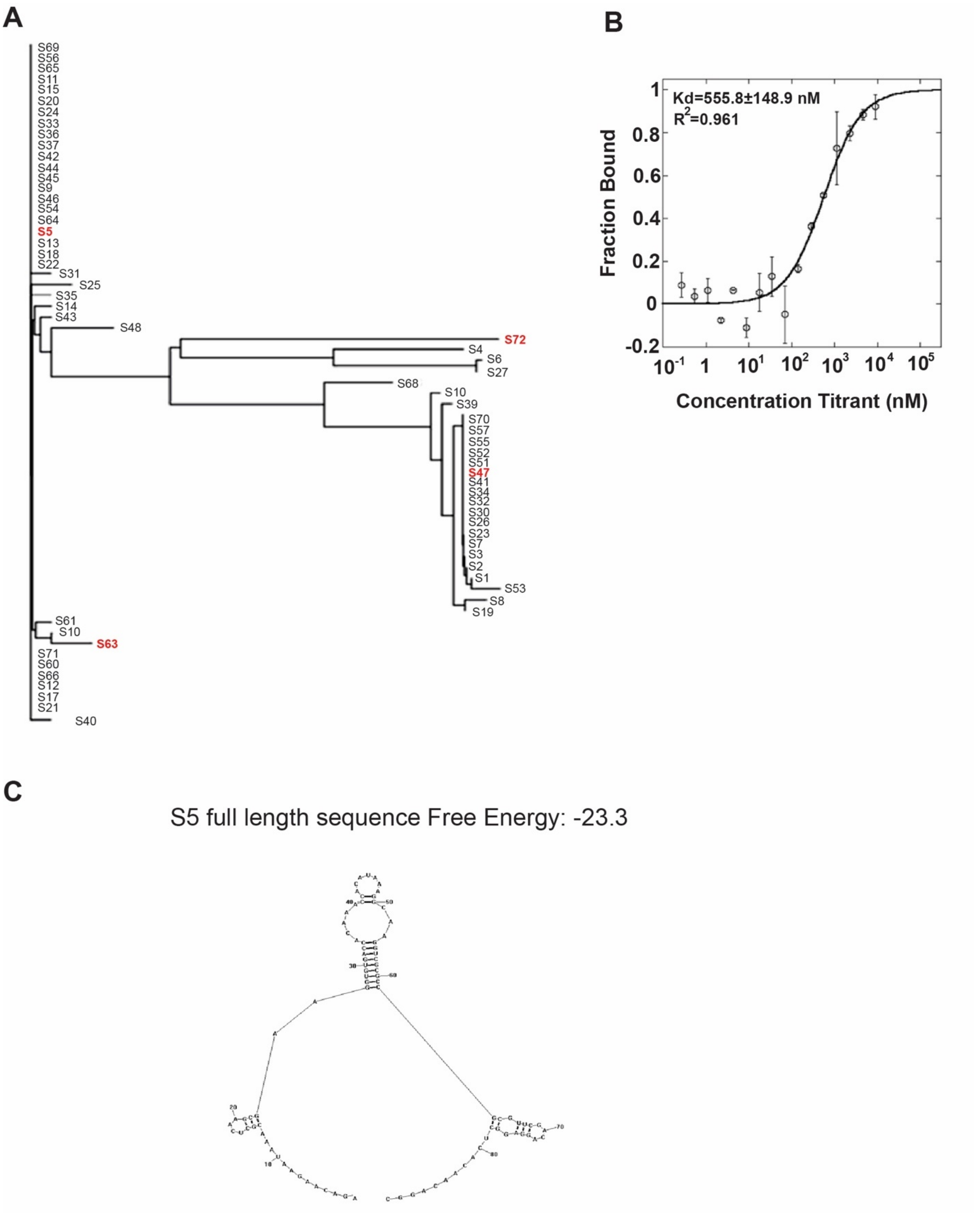

Fig. S14. Phylogenetic tree of the first round of selection and characterization of S5 sequence. (A) Phylogenetic tree generated by using ClustalW2 software and visualized with

TreeVieX program. **(B)** S5 sequence binding curve obtained by microscale thermophoresis (MST). Data represented as mean values ± SD (n = 2 biologically independent experiments). **(C)** Secondary structure prediction of S5 sequence by using RNA structure version 5.1.

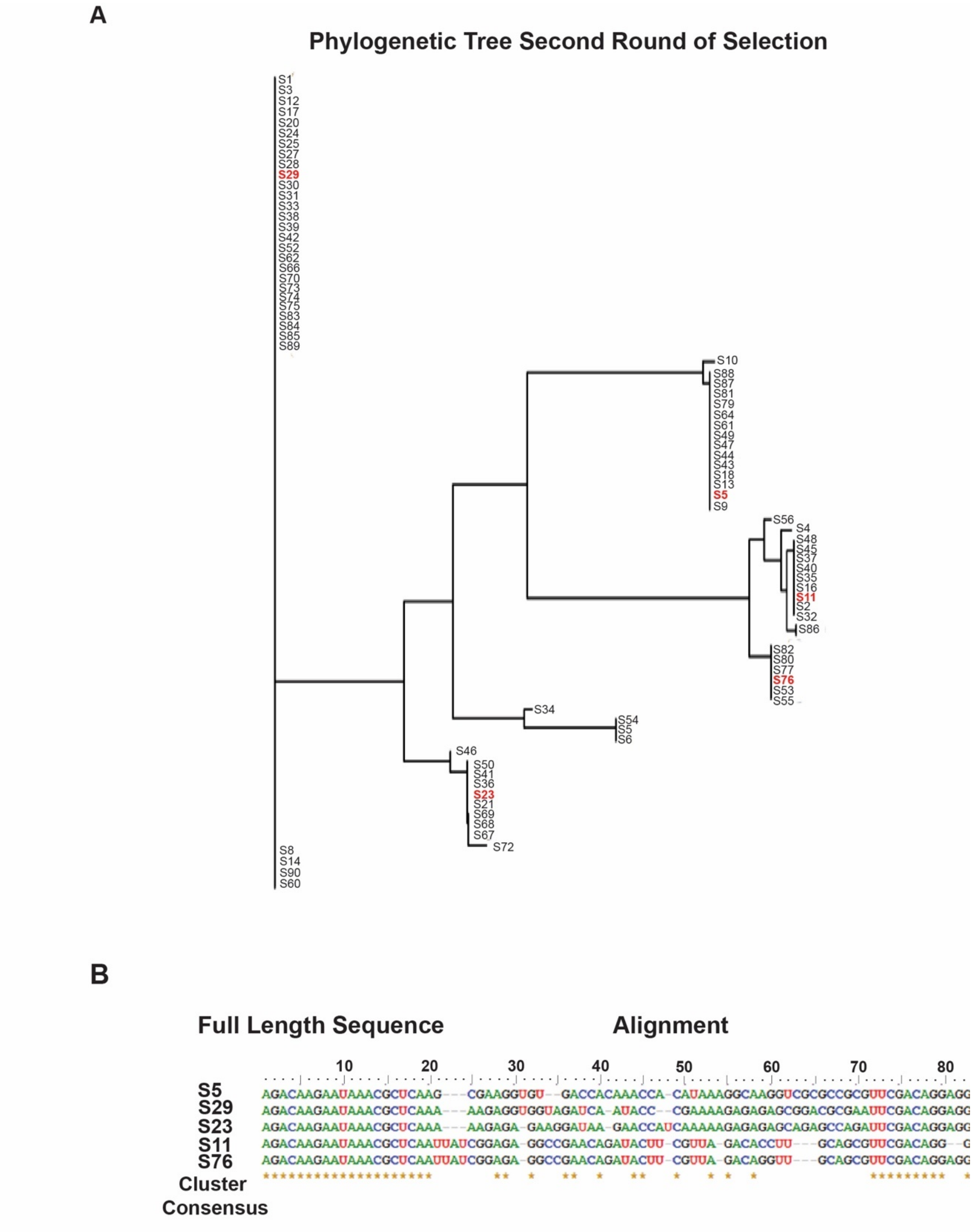

Fig. S15. Phylogenetic tree of the second round of selection and alignment of S5, S11, S23, S29, and S76 sequences. (A) Phylogenetic tree of second round of tandem protein-SELEX. **(B)** Alignment of S5, S11, S23, S29, and S76 sequence.

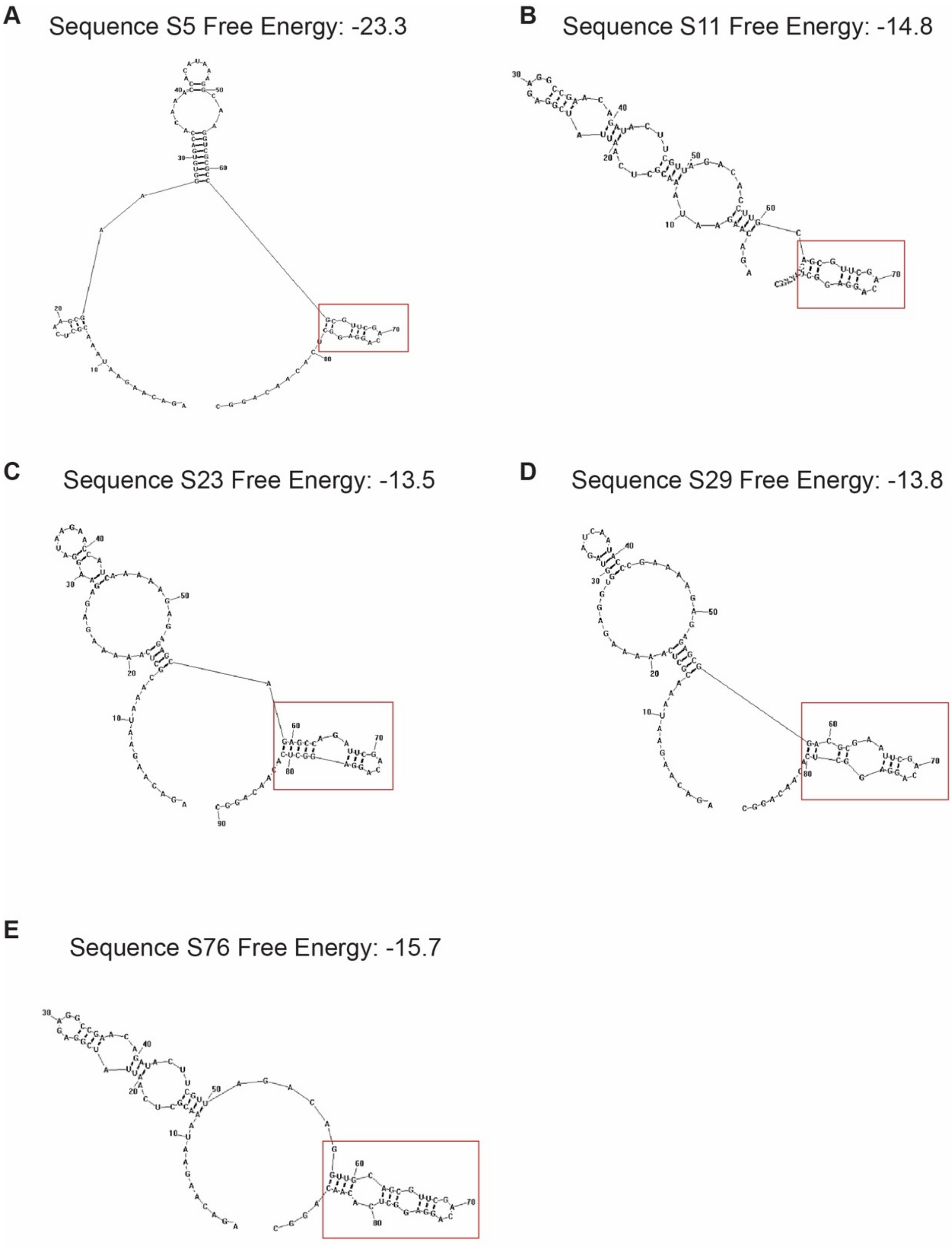

Fig. S16. Identification of binding site for S11, S23, S29, and S76. Prediction of secondary structure of S5 **(A)**, S11 **(B)**, S23 **(C)**, S29 **(D)**, and S76 **(E)** sequences to identify the functional part of the aptamer involved in the recognition of CD5L target.

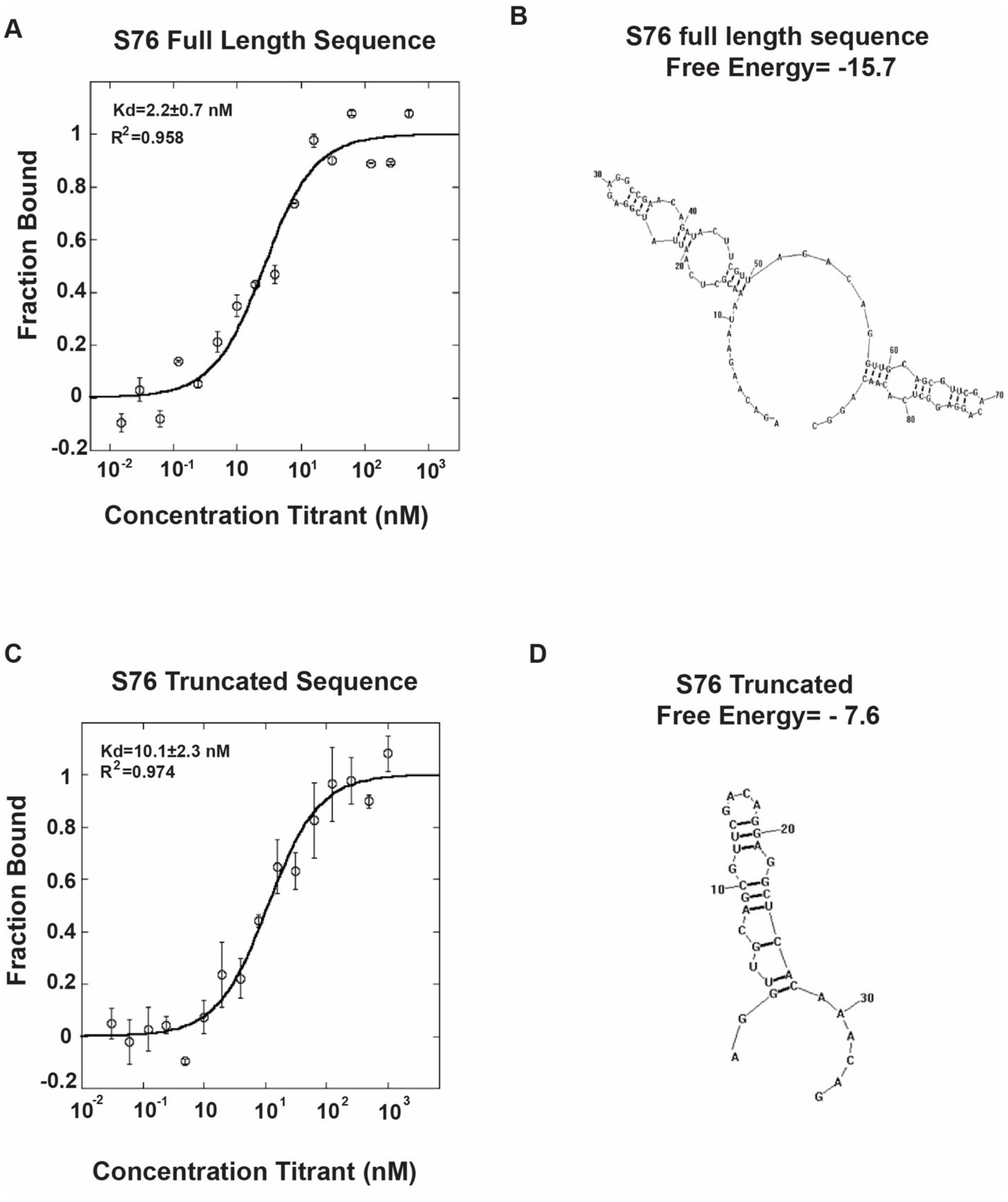

Fig. S17. Comparison of binding affinity and secondary structure of S76 and S76.T. (A, B) Binding affinity (A) of full-length S76 sequence by MST and prediction of secondary structure (B) by using RNA structure version 5.1. **(C, D)** Binding affinity (C) of truncated S76 (S76.T) sequence

by MTS and prediction of secondary structure (D) by using RNA structure version 5.1. For A and C, data represented as mean values ± SD (n = 2 biologically independent experiments).

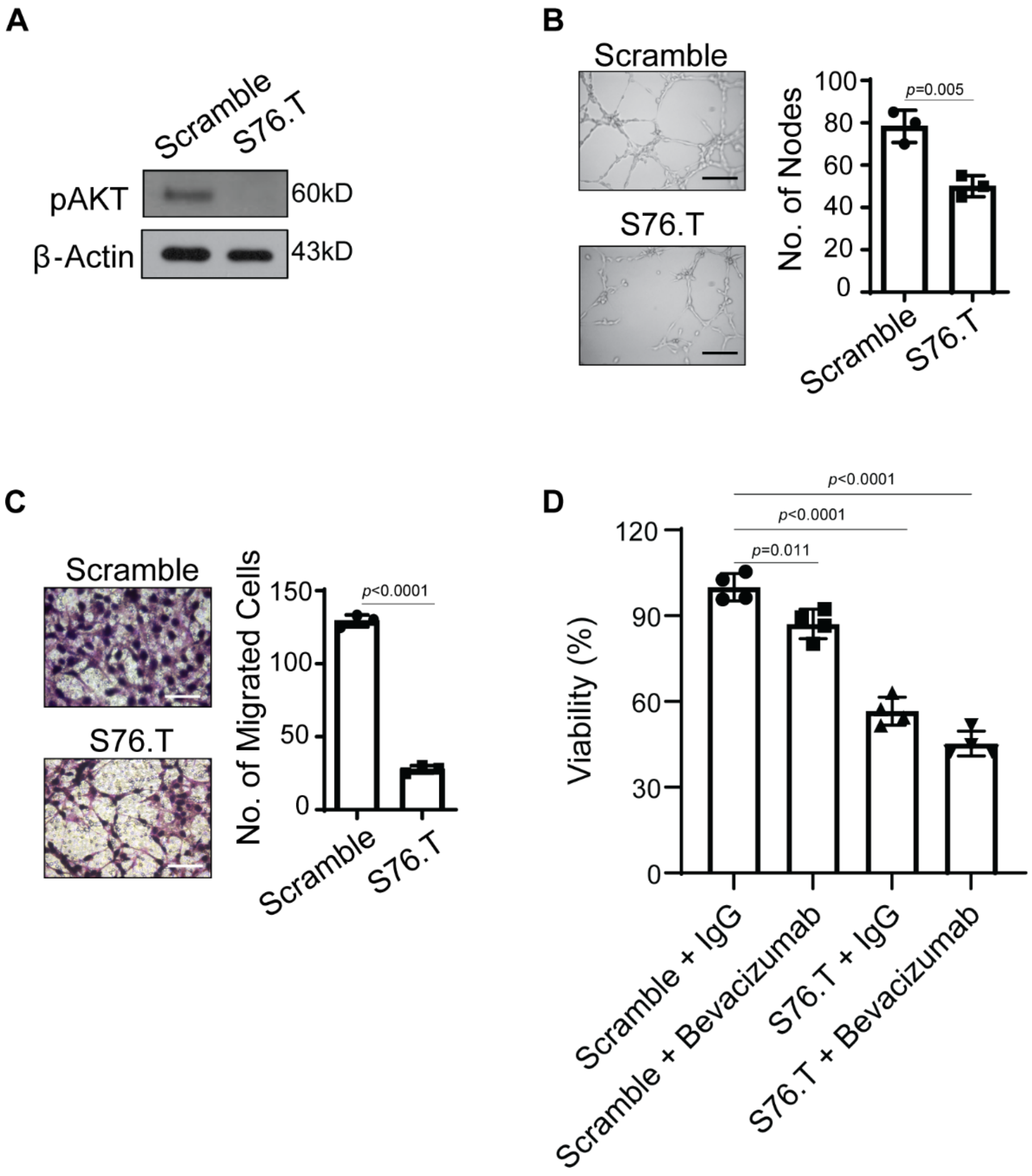

Fig. S18. CD5L aptamer (S76.T) blocks bevacizumab-resistant endothelial cells. (A) Expression of pAKT in bevacizumab-resistant RF24 cells after treatment with the S76.T-aptamer. **(B, C)** S76.T-aptamer significantly reduces tube formation (B) and cell migration (C) compared with scramble aptamer in bevacizumab-resistant RF24 cells (scale bar = 200 μm for both B and C). **(D)** Cell viability assay in RF24 endothelial cells. RF24 endothelial cells were treated with S76.T-aptamer alone or in combination with bevacizumab. Data represented as mean values ± SD, determined by two-tailed Student 's *t* test except one-way Anova Tukey's multiple comparisons for D (n = 3 biologically independent experiments for B, C and n = 4 biologically

independent experiments for D).

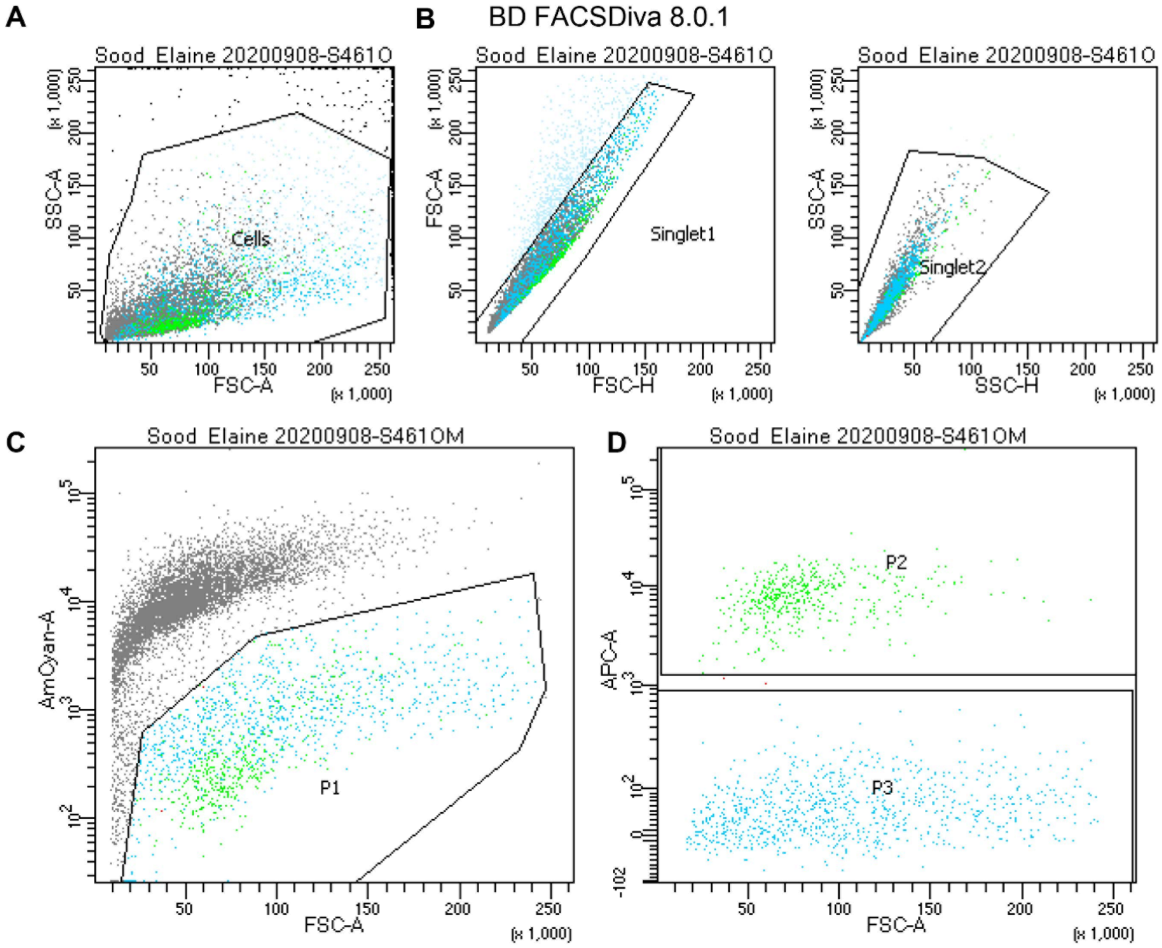

Tube: S461OM

Population	#Events	%Parent	%Total
All Events	10,000	####	100.0
Cells	9,344	93.4	93.4
Singlet1	8,592	92.0	85.9
Singlet2	8,579	99.8	85.8
P1	1,383	16.1	13.8
P2	471	34.1	4.7
P3	910	65.8	9.1

Fig. S19. Representative example of gating strategy for cell sorting applied in single-cell analysis. Cells were first gated by A) All cells; B) single cells / singlets, followed by the cell viability dye; C) where only live cells were gated (AmCyan and FSC-A); and (D) cells were sorted and collected by CD45 positive and CD45 negative cells (APC and FSC-A).

Supplementary Table 1. Kd Binding Values for S5, S47, S63 and S72 Obtained by MTS

Sequence Name	Kd vs CDL5	Kd vs VEGF
S5	555.8±148.9 nM	Weak binding, not fit with the law of mass action
S47	1552.4±309.4 nM	103.4±45.4 nM
S63	No binding between 500 pM to 50 mM	Weak binding, not fit with the law of mass action
S72	Weak binding, not fit with the law of mass action	135.2±61.6 nM

Supplementary Table 2. Kd Binding Values for S11, S23 and S76 Obtained by MTS

Sequence Name	Kd vs CDL5	Kd vs VEGF
S11	59.2±22.14 nM	Weak binding, not fit with the law of mass action
S23	92.1±40.1 nM	Weak binding, not fit with the law of mass action
S76	2.2±0.7 nM	Weak binding, not fit with the law of mass action

Supplementary Table 3. Quantitative-PCR primer lists

No.	Primers	Sequence (5’-3’)
1	Human CD5L F	5'- CTGCTTGTTCTCCTGAGCCC -3'
2	Human CD5L R	5'- TCAAAGGGTCAGGGTTGAGC -3'
3	Human PPAR-γ F	5'- GCCCTTTGGTGACTTTATGGA -3'
4	Human PPAR-γ R	5'- GCAGCAGGTTGTCTTGGATG -3'
5	Human CD36 F	5'- GAGAACTGTTATGGGGCTAT -3'
6	Human CD36 R	5'- TTCAACTGGAGAGGCAAAGG -3'
7	Tie2 Cre transgene F	5'-CGCATAACCAGTGAAACAGCATTGC-3'
8	Tie2 Cre transgene R	5'-CCCTGTGCTCAGACAGAAATGAGA-3'

Supplemental Table 4. Materials used

Reagent or resource	Source	Identifier
**Experimental models: cell lines**		
SKOV3ip1	MDA Cell Line Core	CVCL_0C84
ID8	Dr. Roby at Univ. of Kansas	CVCL_IU14
RF24	ABM (Applied Biological Materials)	CVCL_AX74
HEK293T	ATCC	CVCL_0063
HPAEC	ATCC	N/A
HUVEC-GFP	ANGIO-PROTEOMIE	CAP-0001GFP
**Experimental models: organisms/strains**		
Mouse: *PPARG*$^{fl/fl}$;*Tie*$^{2-}$*Cre*$^{+/-}$ female and male	Dr. Yihong Wan, University of Texas Southwestern (10 mice/group)	N/A
Mouse: Female athymic nude mice (NCr-nu)	Taconic (10 mice/group)	N/A
**Chemicals, peptides, and recombinant proteins**		
Optimal Cutting Temperature (OCT) media	Miles, Inc., Elkhart, IN	Catalog no. 25608-930
RNeasy mini kit	Qiagen, Hilden, Germany	Catalog no. 74104
SYBR Green ER qPCR SuperMix Universal	Invitrogen, Carlsbad, CA	Catalog no. 4368708
siRNAs	Sigma-Aldrich, St. Louis, MO	Universal negative control; SIC001<br>*CD5L:* SASI_Hs02_00340494<br>*PPARG:* SASI_Hs01_00106498
Lipofectamine 2000	Invitrogen	Catalog no. 11668027
LY294002	Selleckchem, Houston, TX	Catalog no. S1105
Topotecan	Sigma-Aldrich	123948-87-8

YC-1	Sigma-Aldrich	170632-47-0
Cobalt Chloride, $CoCl_2$	Sigma-Aldrich	7646-79-9
Human recombinant CD5L protein	Sino Biological, Beijing, China	Catalog no. 10791-H08H
Bevacizumab	Genentech, South San Francisco, CA	N/A
MTT reagent	Sigma-Aldrich	Catalog no. M2128
Aptamer	Sigma-Aldrich	N/A
His-tagged VEGF-A	Abnova, Taiwan	Catalog no. P5816
His-tagged CD19	Life Technologies, Carlsbad, CA	Catalog no. 11880H08H250
6-His Peptide tagged	BioLegend, San Diego, CA	Catalog no. 931601
DNA library	TriLink Biotechnologies, San Diego, CA	Fitzwater T. and Polisky B. (1996)
M-MuLV Reverse Transcriptase	Roche, Indianapolis, IN	Catalog no. 11062603001
Ni^{2+} NTA Magnetic Agarose Beads	Qiagen	Catalog no. 36111
TRIzol Reagent	Life Technologies	Catalog no. 15596026
TOPO Cloning	Life Technologies	Catalog no. K4500-01SC, K4550-01SC, K4600-01SC
**Antibodies: Expressed in HEK293 suspension cells and purified with use of protein A affinity resin to >95% purity.**		
CD31	Cell Signaling Technologies, Beverly, MA	Catalog no. 77699, RRID:AB_2722705 (1:100 dilution)
PE-CD31	BD Biosciences, San Jose, CA	Catalog no. 555446, RRID:AB_395839 (1:200 dilution)
CD31	Pharmingen, San Diego, CA	Catalog no. 557355, RRID:AB_396660 (1:800 dilution)
Ki67	Neomarkers, Fremont, CA	Catalog no. RM-9106-R7, RRID:AB_149920 (1:200 dilution)

AKT	Cell Signaling Technologies	Catalog no. 4691, RRID:AB_915783<br>Catalog no. 9272, RRID:AB_329827;<br>(1:1000 dilution)
Phospho-AKT	Cell Signaling Technologies	Catalog no. 4060, RRID:AB_2315049<br>(1:000 dilution)
Phospho-AKT	Abcam, Cambridge, MA	Catalog no. ab81283<br>RRID:AB_2224551<br>(1:50 dilution)
PPARG	Cell Signaling Technologies	Catalog no. 2430, RRID:AB_823599<br>(1:200 dilution)
PPARG	Abcam	Catalog no. ab59256,<br>RRID:AB_944767<br>(1:1000 dilution)
HIF1α	Thermo Fisher Scientific, Waltham, MA	Catalog no. MA1-516, RRID:AB_325431<br>(1:1000 dilution)
CD5L (AIM)	Santa Cruz Biotechnology, Santa Cruz, CA<br>R&D Systems, Minneapolis, MN	Catalog no. sc-514281;<br>RRID:AB_2076351<br>(1:1000 dilution)
CD5L (AIM)	Invitrogen, Carlsbad, CA	Catalog no. 703558, RRID:AB_2762393<br>(1:200 dilution)
CD5L (AIM)	R&D Systems, Minneapolis, MN	Catalog no. AF2797, RRID:AB_2076351<br>(1:500 dilution)
CD5L (AIM)	Thermo Fisher Scientific, Waltham, MA	Catalog no. PA5-84779, RRID:AB_2791929<br>(0.04-0.4 μg/mL)
CD36	Abcam, Cambridge, MA	Catalog no. ab252922<br>(1:1000 dilution)
β-actin	Sigma-Aldrich	Catalog no. A5441, RRID:AB_476744<br>(1:5000 dilution)
Vinculin	Sigma-Aldrich	Catalog no. V9131, RRID:AB_477629<br>(1:2000 dilution)
Anti-rabbit secondary	Sigma-Aldrich	Catalog no. NA934, RRID:AB_772206

antibodies conjugated with horseradish peroxidase.		(1:2000 dilution)
Anti-mouse secondary antibodies conjugated with horseradish peroxidase.	Sigma-Aldrich	Catalog no. NA931, RRID:AB_772210<br>(1:2000 dilution)
pLenti-C-mGFP-human CD5L vector	OriGene, Rockville, MD	Catalog no. RC206528L2
**Critical commercial assays**		
Click-iT EdU Assay Kit	Invitrogen	Catalog no. C10632
CD5L ELISA Kit	Mybiosource, San Diego, CA, USA	Catalog no. MBS2024653
Verso cDNA kit	Thermo Scientific	Catalog no. AB1453B
RNeasy mini kit	Qiagen	Catalog no. 74106
EZ ChIP™ kit	Millipore, Temecula, CA	Catalog no. 17-371
**Software and algorithms**		
GraphPad Prism 7.0.	GraphPad Prism 7.0.	GraphPad Software, La Jolla, CA
Windows statistical software	SPSS version 12 for Windows statistical software	SPSS, Inc., Chicago, IL
R statistical package	R.3.4.1	R Foundation for Statistical Computing, Vienna, Austria
ImageJ	ImageJ 1.52a	Wayne Rasband, National Institutes of Health, Bethesda, MD
ClustalW2 software	http://bonsai.hgc.jp/~mdehoon/software/cluster/software.htm	de Hoon M.J.<br>Imoto S.<br>Nolan J.<br>Miyano S.<br>Open source clustering software.<br>Bioinformatics. 2004; 20: 1453-1454
TreeViewX	http://jtreeview.sourceforge.net/	Saldanha A.J.

program		Java Treeview--extensible visualization of microarray data. Bioinformatics. 2004; 20: 3246-3248
Monolith NT.115	NanoTemper Technologies GmbH, Munich, Germany	Stoltenburg R et al. PLoS One. 2015
