# Supplementary material for: Overcoming adaptive resistance to anti-VEGF therapy by targeting CD5L
Source: Nat Commun. 2023 Apr 26;14:2407. doi: 10.1038/s41467-023-36910-5 (PMC10133315; doi:10.1038/s41467-023-36910-5)
Supplement: Supplementary file 3 — Reporting Summary [file 41467_2023_36910_MOESM3_ESM.pdf]

nature portfolio

Corresponding author(s): Yunfei Wen, Anil K. Sood

Last updated by author(s): Feb 14, 2023

Reporting Summary

Nature Portfolio wishes to improve the reproducibility of the work that we publish. This form provides structure for consistency and transparency in reporting. For further information on Nature Portfolio policies, see our Editorial Policies and the Editorial Policy Checklist.

Statistics

For all statistical analyses, confirm that the following items are present in the figure legend, table legend, main text, or Methods section.

n/a	Confirmed	
☐	☒	The exact sample size (*n*) for each experimental group/condition, given as a discrete number and unit of measurement
☐	☒	A statement on whether measurements were taken from distinct samples or whether the same sample was measured repeatedly
☐	☒	The statistical test(s) used AND whether they are one- or two-sided<br>*Only common tests should be described solely by name; describe more complex techniques in the Methods section.*
☒	☐	A description of all covariates tested
☐	☒	A description of any assumptions or corrections, such as tests of normality and adjustment for multiple comparisons
☐	☒	A full description of the statistical parameters including central tendency (e.g. means) or other basic estimates (e.g. regression coefficient) AND variation (e.g. standard deviation) or associated estimates of uncertainty (e.g. confidence intervals)
☐	☒	For null hypothesis testing, the test statistic (e.g. F, t, r) with confidence intervals, effect sizes, degrees of freedom and P value noted<br>*Give P values as exact values whenever suitable.*
☒	☐	For Bayesian analysis, information on the choice of priors and Markov chain Monte Carlo settings
☒	☐	For hierarchical and complex designs, identification of the appropriate level for tests and full reporting of outcomes
☐	☒	Estimates of effect sizes (e.g. Cohen's *d*, Pearson's *r*), indicating how they were calculated

Our web collection on statistics for biologists contains articles on many of the points above.

Software and code

Policy information about availability of computer code

Data collection	1) Leica inverted LED Fluorescence Motorized Microscope was used to collect IHC and other images; 2) BD FACSAria was used for acquiring flow cytometry data; 3) IVIS Spectrum small animal imaging was used to collect and analysis the bio-luminescence signals.
Data analysis	(1) Graph Pad Prism v7.0, (2) SPSS version 12 for Windows statistical software, (3) ImageJ 1.52a, 4) ClustalW2 software,5)TreeViewX

For manuscripts utilizing custom algorithms or software that are central to the research but not yet described in published literature, software must be made available to editors and reviewers. We strongly encourage code deposition in a community repository (e.g. GitHub). See the Nature Portfolio guidelines for submitting code & software for further information.

Data

Policy information about availability of data

All manuscripts must include a data availability statement. This statement should provide the following information, where applicable:

- Accession codes, unique identifiers, or web links for publicly available datasets
- A description of any restrictions on data availability
- For clinical datasets or third party data, please ensure that the statement adheres to our policy

The single cell data was submitted to GEO under the accession number GSE181955. The microarray data was submitted to GEO under the accession number GSE180687.

Human research participants

Policy information about studies involving human research participants and Sex and Gender in Research.

Reporting on sex and gender	N/A
Population characteristics	N/A
Recruitment	N/A
Ethics oversight	N/A

Note that full information on the approval of the study protocol must also be provided in the manuscript.

Field-specific reporting

Please select the one below that is the best fit for your research. If you are not sure, read the appropriate sections before making your selection.

[x] Life sciences [ ] Behavioural & social sciences [ ] Ecological, evolutionary & environmental sciences

For a reference copy of the document with all sections, see nature.com/documents/nr-reporting-summary-flat.pdf

Life sciences study design

All studies must disclose on these points even when the disclosure is negative.

Sample size	For the animal experiments in Figure 4 and 6, 10 mice were assigned per treatment group. This sample size gave 80% power to detect a 50% reduction in tumor weight with a 95% confidence interval. Since the in vivo experiment in Figure 5 was designed to screen antibody candidates, only 7 mice were assigned per treatment group.
Data exclusions	No data were excluded from analysis
Replication	Except for the in vivo studies, at least 3 independent samples were used for each analysis in each cell-based experiments; Western blots were performed in two independent technical replicates. All attempts at replication showed consistent results.
Randomization	For the in vivo studies, animals were assigned randomly to experimental and control groups.
Blinding	All group allocations were blinded in all in vivo experiments. Individuals who performed the necropsy were blinded to the treatment group assignments in all animal experiments.

Reporting for specific materials, systems and methods

We require information from authors about some types of materials, experimental systems and methods used in many studies. Here, indicate whether each material, system or method listed is relevant to your study. If you are not sure if a list item applies to your research, read the appropriate section before selecting a response.

Materials & experimental systems

n/a	Involved in the study	
[ ]	[x]	Antibodies
[ ]	[x]	Eukaryotic cell lines
[x]	[ ]	Palaeontology and archaeology
[ ]	[x]	Animals and other organisms
[ ]	[x]	Clinical data
[x]	[ ]	Dual use research of concern

Methods

n/a	Involved in the study	
[x]	[ ]	ChIP-seq
[ ]	[x]	Flow cytometry
[x]	[ ]	MRI-based neuroimaging

Antibodies

Antibodies used	All antibodies used in this study have been listed in Supplemental Table 4 with RRID.<br>CD31 Cell Signaling Technologies, Beverly, MA Catalog no. 77699, RRID:AB_2722705<br>(1:100 dilution)<br>PE-CD31 BD Biosciences, San Jose, CA Catalog no. 555446, RRID:AB_395839

(1:200 dilution)
CD31 Pharmingen, San Diego, CA Catalog no. 557355, RRID:AB_396660
(1:800 dilution)
Ki67 Neomarkers, Fremont, CA Catalog no. RM-9106-R7, RRID:AB_149920
(1:200 dilution)
AKT Cell Signaling Technologies Catalog no. 4691, RRID:AB_915783 Catalog no. 9272, RRID:AB_329827;
(1:1000 dilution)
Phospho-AKT Cell Signaling Technologies
Catalog no. 4060, RRID:AB_2315049
(1:000 dilution)
Phospho-AKT Abcam, Cambridge, MA Catalog no. ab81283
RRID:AB_2224551
(1:50 dilution)
PPARG Cell Signaling Technologies Catalog no. 2430, RRID:AB_823599
(1:200 dilution)
PPARG Abcam Catalog no. ab59256,
RRID:AB_944767
(1:1000 dilution)
HIF1α Thermo Fisher Scientific, Waltham, MA Catalog no. MA1-516, RRID:AB_325431
(1:1000 dilution)
CD5L (AIM) Santa Cruz Biotechnology, Santa Cruz, CA
R&D Systems, Minneapolis, MN Catalog no. sc-514281;
RRID:AB_2076351 (1:1000 dilution)
CD5L (AIM) Invitrogen, Carlsbad, CA Catalog no. 703558, RRID:AB_2762393
(1:200 dilution)
CD5L (AIM) R&D Systems, Minneapolis, MN Catalog no. AF2797, RRID:AB_2076351
(1:500 dilution)
CD5L (AIM) Thermo Fisher Scientific, Waltham, MA Catalog no. PA5-84779, RRID:AB_2791929
(0.04-0.4 μg/mL)
CD36 Abcam, Cambridge, MA Catalog no. ab252922
(1:1000 dilution)
β-actin Sigma-Aldrich Catalog no. A5441, RRID:AB_476744
(1:5000 dilution)
Vinculin Sigma-Aldrich Catalog no. V9131, RRID:AB_477629
(1:2000 dilution)
Anti-rabbit secondary antibodies conjugated with horseradish peroxidase. Sigma-Aldrich Catalog no. NA934, RRID:AB_772206
(1:2000 dilution)
Anti-mouse secondary antibodies conjugated with horseradish peroxidase. Sigma-Aldrich Catalog no. NA931, RRID:AB_772210
(1:2000 dilution)

Validation

1. anti-CD31 has been validated to be used for immunohistochemistry and mentioned species reactivity with mouse (https://www.cellsignal.com/products/primary-antibodies/cd31-pecam-1-d8v9e-xp-rabbit-mab/77699).
2. PE-anti-CD31 has been validated to be used for immunohistochemistry and mentioned species reactivity with human (https://www.bdbiosciences.com/en-us/products/reagents/flow-cytometry-reagents/research-reagents/single-color-antibodies-ruo/pe-mouse-anti-human-cd31.555446).
3. anti-CD31 has been validated to be used for flow cytometric analysis and immunohistochemistry and mentioned species reactivity with mouse (https://www.bdbiosciences.com/en-eu/products/reagents/flow-cytometry-reagents/research-reagents/single-color-antibodies-ruo/purified-rat-anti-mouse-cd31.557355).
4. anti-Ki67 has been validated to be used for immunohistochemistry and mentioned species reactivity with mouse and human (https://www.thermofisher.com/antibody/product/MA5-14520.html?CID=AFLLO-MA5-14520). * Neomarkers is now a part of invitrogen
5. anti-AKT has been validated to be used for western blotting analysis mentioned species reactivity with mouse and human (https://www.cellsignal.com/products/primary-antibodies/akt-pan-c67e7-rabbit-mab/4691).
6. anti-AKT has been validated to be used for western blotting analysis mentioned species reactivity with mouse and human (https://www.cellsignal.com/products/primary-antibodies/akt-antibody/9272).
7. anti-phospho-AKT(Ser473) has been validated to be used for western blotting analysis mentioned species reactivity with mouse and human (https://www.cellsignal.com/products/primary-antibodies/phospho-akt-ser473-d9e-xp-rabbit-mab/4060).
8. anti-phospho-AKT1(Ser473) has been validated to be used for immunohistochemistry and mentioned species reactivity with mouse and human (https://www.abcam.com/akt1-phospho-s473-antibody-ep2109y-ab81283.html).
9. anti-PPARG has been validated to be used for western blotting analysis and immunohistochemistry and mentioned species reactivity with mouse and human (https://www.cellsignal.com/products/primary-antibodies/pparg-d69-antibody/2430).
10. anti-PPARG has been validated to be used for western blotting analysis and mentioned species reactivity with human (https://www.abcam.com/ppar-gamma-antibody-ab59256.html).
11. anti-HIF1α has been validated to be used for western blotting analysis and mentioned species reactivity with human (https://www.thermofisher.com/antibody/product/HIF1A-Antibody-clone-mgc3-Monoclonal/MA1-516).
12. anti-CD5L (AIM) has been validated to be used for western blotting analysis and mentioned species reactivity with human (https://www.scbt.com/p/cd5l-antibody-f-10).
13. anti-CD5L (AIM) has been validated to be used for immunohistochemistry and mentioned species reactivity with mouse (https://www.thermofisher.com/antibody/product/AIM-Antibody-clone-11H48L16-Recombinant-Monoclonal/703558).

14. anti-CD5L (AIM) has been validated to be used for immunohistochemistry and mentioned species reactivity with human (https://www.rndsystems.com/products/human-cd5l-antibody_af2797).
15. anti-CD5L (AIM) has been validated to be used for western blotting analysis and mentioned species reactivity with human (https://www.thermofisher.com/antibody/product/AIM-Antibody-Polyclonal/PA5-84779).
16. anti-CD36 has been validated to be used for western blotting analysis and mentioned species reactivity with human (https://www.abcam.com/cd36-antibody-epr22509-40-ab252922.html).
17. anti-β-actin has been validated to be used for western blotting analysis and mentioned species reactivity with mouse and human (https://www.sigmaaldrich.com/US/en/product/sigma/a5441).
18. anti-Vinculin has been validated to be used for western blotting analysis and mentioned species reactivity with mouse and human (https://www.sigmaaldrich.com/US/en/product/sigma/v9131).
19. anti-rabbit secondary antibodies conjugated with horseradish peroxidase has been validated to be used for western blotting analysis and mentioned species reactivity with rabbit (https://www.sigmaaldrich.com/US/en/product/sigma/sab3700934).
20. anti-mouse secondary antibodies conjugated with horseradish peroxidase has been validated to be used for western blotting analysis and mentioned species reactivity with mouse (https://www.sigmaaldrich.com/US/en/product/sigma/sab3701095).

Eukaryotic cell lines

Policy information about cell lines and Sex and Gender in Research

Cell line source(s)	SKOV3ip1 MDA Cell Line Core CVCL_0C84<br>ID8 Dr. Roby at Univ. of Kansas CVCL_IU14<br>RF24 ABM (Applied Biological Materials) CVCL_AX74<br>HEK293T ATCC CVCL_0063<br>HPAEC ATCC N/A<br>HUVEC-GFP ANGIO-PROTEOMIE CAP-0001GFP
Authentication	Cell lines were obtained from the MD Anderson Cytogenetics and Cell Authentication Core (CCAC), which supplies authenticated cell lines through Short Tandem Repeat (STR) DNA profiling, which is based on screening regions of microsatellite instability with defined tri- or tetrad-nucleotide repeats located throughout the chromosomes. PCR reactions using primers on non-repetitive flanking those regions will generate PCR products of different sizes based on the number of repeats in the region; the size of these PCR products is determined by capillary electrophoresis. By combining between 8 and 16 STR loci, such as D5S818, D13S317, D7S820, D16S539, vWA, TH01, TPOX, and CSF1PO, it is possible to uniquely identify a sample. The CCLC assay screens 16 loci using the Promega Powerplex 16 HS kit. The CCLC test also includes matching the STR profiles against an internal database comprised of public profiles and profiles that are unique to cell lines developed by MDACC investigators.
Mycoplasma contamination	Mycoplasma determination was performed in each cell line periodically using Lonza MycoAlert Kit.
Commonly misidentified lines<br>(See ICLAC register)	No cell line included in this study is listed as commonly misidentified cell lines.

Animals and other research organisms

Policy information about studies involving animals; ARRIVE guidelines recommended for reporting animal research, and Sex and Gender in Research

Laboratory animals	Female athymic nude mice (NCr-nu) and C57BL/6 (B6-F) wild-type mice were purchased from the Taconic Biosciences (Rensselaer, NY) and maintained as described previously22. All mouse studies were approved by the Institutional Animal Care and Use Committee. Mice were cared for in accordance with guidelines set forth by the American Association for Accreditation of Laboratory Animal Care and the US Public Health Service Policy on Humane Care and Use of Laboratory Animals.
Wild animals	This study did not involve wild animals.
Reporting on sex	Only female mice are used in this study for purpose of the establishment of orthotopic ovarian cancer models.
Field-collected samples	This study did not involve samples collected from field.
Ethics oversight	All mouse studies were approved by the Institutional Animal Care and Use Committee. Mice were cared for in accordance with guidelines set forth by the American Association for Accreditation of Laboratory Animal Care and the US Public Health Service Policy on Humane Care and Use of Laboratory Animals (Protocol number 00001029)

Note that full information on the approval of the study protocol must also be provided in the manuscript.

Clinical data

Policy information about clinical studies

All manuscripts should comply with the ICMJEguidelines for publication of clinical research and a completedCONSORT checklist must be included with all submissions.

Clinical trial registration	N/A
Study protocol	N/A
Data collection	N/A
Outcomes	N/A

Flow Cytometry

Plots

Confirm that:

- [x] The axis labels state the marker and fluorochrome used (e.g. CD4-FITC).
- [x] The axis scales are clearly visible. Include numbers along axes only for bottom left plot of group (a 'group' is an analysis of identical markers).
- [x] All plots are contour plots with outliers or pseudocolor plots.
- [x] A numerical value for number of cells or percentage (with statistics) is provided.

Methodology

Sample preparation	Fresh tissue was obtained from the MD Anderson Tumor bank and sample processing was done right after tissue collection from the patient. About 2 grams of tissue were minced and dissociated using Accumax™. After dissociation, cells were washed with complete media to stop the reaction, centrifuged and resuspended in freeze media (90% FBS/ 10% DMSo) and frozen at -80 C. For flow cytometry, cells were thawed at 37C and centrifuged. After that, cells were resuspended in FACS buffer with 1:100 Fc Block and LIDE/DEAD aqua and incubated for 30min in ice. Cell were then washed in FACS buffer and resuspended in the same solution buffer.
Instrument	BD FACSAria™
Software	We did not perform further analysis on the flow cytometry data. The flow was solely used to sort live cells for single cell analysis.
Cell population abundance	1 gram of tissue in single cell solution per sample
Gating strategy	Cells were gated by singlets and by LIVE/DEAD Aqua. The cells negative for the staining (live cells) were sorted for further single cell analysis.

- [x] Tick this box to confirm that a figure exemplifying the gating strategy is provided in the Supplementary Information.
