# Supplementary material for: Overcoming adaptive resistance to anti-VEGF therapy by targeting CD5L
Source: Nat Commun. 2023 Apr 26;14:2407. doi: 10.1038/s41467-023-36910-5 (PMC10133315; doi:10.1038/s41467-023-36910-5)
Supplement: Supplementary file 4 — Source Data [file 41467_2023_36910_MOESM4_ESM.pdf]

Fig. 1A

Photon counts E+8 value

day	Control Ig	Control Ig	Control Ig	Control Ig	Control IgG #5		B20 #1	B20 #2	B20 #3	B20 #4	B20 #5	B20 #6	B20 #7	B20 #8	B20 #9	B20 #10
18	2.2	2.1	1.9	1.9	2		1.9	1.7	2.4	2.7	2.1	2.5	2.4	2.2	1.6	2
23	2.5	2.4	2.1	2	2.5		1.7	1.7	2	2.3	1.8	2.2	2.1	1.7	1.6	1.7
27	3.5	3.2	3.1	2.9	3.6		1.4	1.3	1.8	1.8	1.5	1.8	1.7	1.4	1.7	1.6
30	5	5.5	6.5	5	6.1							1.5	1.6	1.4	1.5	1.5
33												1.7	1.8	1.8	2.1	1.7
37												2	2.12	2.5	2.7	1.6
41												3.5	3.3	3.1	4.1	2.9
45												5	5.3	5.7	6	4.2
48												6.5	6.8	7.1	8.6	5.6

Fig. 1E

EDU (Cell proliferation)

EV	CD5L
5.02	7.92
4.24	5.24
3.28	5.86

Fig. 1F

Tube formation

EV	CD5L
45	88
51	82
48	80
	76

Fig. 1G

Cell migration

EV	CD5L
66	132
74	120
61	133
60	116

Fig. 1H

CD5L ELISA

EV	CD5L
9.260241	184.8653
7.880931	195.649

Fig. 1J

EDU (Cell proliferation)

siControl	siCD5L
4	3
4.5	2.85
5	2.72

Fig. 1K

Tube formation

siControl	siCD5L
68	38
61	42
70	40
63	35

Fig. 1L

Cell migration

siControl	siCD5L
63	35
60	33
71	41
60	40

Fig. 2A

CD5L

No insert	PPARG
0.93080	9.26337
1.06920	9.07273
0.96882	8.99609
1.03118	9.70884

Fig. 2C

Promoter assay(Relative)

EV	PPARG
1.06312	4.05578
0.94396	4.33252
1.25944	6.75979
0.82919	4.80901

Fig. 2D
mRNA expression

PPARG		CD5L	
**siControl**	**siPPARG**	**siControl**	**siPPARG**
0.947485521	0.077598675	0.962814624	0.345158277
1.008475337	0.111273027	0.976255016	0.58858798
1.044039143	0.103821405	1.06093036	0.372504559

Fig. 2F

Promoter assay

siControl	siPPARG
112.53086	15.27833
89.98026	14.83447
124.70185	53.32225
92.68897	41.11331
89.34872	63.56311

Fig. 2G

Promoter assay

pCD5L WT	pCD5L Del
110.21458	2.4962
87.11254	2.038
91.62548	1.74

Fig. 2H

mRNA expression

PPARG		CD5L	
**Normoxia**	**hypoxia**	**Normoxia**	**hypoxia**
1	2.873727	1	7.625459
1	3.182846	1	7.348059
1	3.000672	1	4.640300

Fig. 2J

mRNA expression

PPARG			CD5L		
**0h**	**6h**	**30h**	**0h**	**6h**	**30h**
1	1.121344	6.799305	1	2.576497	14.62047
1	1.198064	7.552638	1	0.887831	12.80429
1	0.824690	7.585616			

Fig. 2L

mRNA expression

PPARG			CD5L		
**DMSO**	**YC-1**	**Topotecan**	**DMSO**	**YC-1**	**Topotecan**
1.016043	0.173711	0.105249	0.979598	0.034266	0.012674
0.983957	0.153546	0.104433	1.020402	0.032612	0.010671
1.000651	0.280981	0.276226			
0.999349	0.253919	0.278437			

Fig. 2M

Promoter assay

Normoxia	Hypoxia
0.920106	60.45985
1.194318	51.96905
0.885577	46.73146

Fig. 2N

ChIP assay

	Normoxia			Hypoxia		
Region1	1.0000	1.0000	1.0000	82.5203	98.8969	76.9284
Region2	3.1571	2.8898	2.2373	1.2704	1.2624	1.2657
Region3	0.7715	0.6343	0.6264	2.0719	1.7716	1.7836

Fig. 3C

Tube formation

DMSO	LY294002
125	65
120	75
131	75
	71

Fig. 3D

Cell migration

DMSO	LY294002
110	72
95	84
104	70

Fig. 3E

CD36 expression

Control	CD5L
1.011485	2.768798
0.9770301	2.098356
1.011485	2.461026

Fig. 3G

Viability(%)

	**RF24/control**				**RF24/CD5L**			
Bev 0	100.0	100.0	100.0	100.0	100.0	100.0	100.0	100.0
Bev 0.25	78.0	75.0	62.5	71.1	95.0	96.0	101.0	99.7
Bev 1	65.0	68.0	69.1	60.4	91.0	89.0	95.0	82.5

Fig. 3H

Viability(%)

	RF24/siControl				RF24/siCD5L			
Bev 0	100.0	100.0	100.0	100.0	100.0	100.0	100.0	100.0
Bev 0.25	80.0	91.0	78.0	75.9	72.6	75.0	62.1	73.0
Bev 1	64.5	67.1	60.4	77.0	51.2	55.0	49.7	51.0

Fig. 4B

Tumor weight	
**WT**	**PPARG KO**
0.89	0.42
0.93	0.43
0.84	0.61
1.93	0.41
0.92	0.53

Fig. 4C

Tumor nodules	
**WT**	**PPARG KO**
28	15
22	16
25	18
38	12
32	20

Fig. 4D

Ki67	
**WT**	**PPARG KO**
60	20
64	30
55	35
70	

Fig. 4E

CD31	
**WT**	**PPARG KO**
30	20
35	10
30	12

Fig. 4F

Survival

Days	**WT**	**PPARG KO**
0	100	100
40	66.667	
41	33.333	80
42		
49	0	
55		60
60		42
62		20
78		0

Fig. 5B

Tumor weight(g)

Con Ab (n=13)	H-447 (n=7)	R-35 (n=7)
0.06	0	0.06
1.05	0	0
1.81	0	0
1.01	0	0.09
0.08	0.39	0
0	0	0.41
0.14	0	0
0.698		
0.02		
0.15		
0.1		
1.66		
0		

Fig. 5C

Tumor nodules #

Con Ab (n=13)	H-447 (n=7)	R-35 (n=7)
2	0	3
40	0	0
22	0	0
32	0	2
5	15	0
0	0	9
16	0	0
15		
2		
10		
3		
25		
0		

Fig. 5D

CD31

Cont Ab	CD5L Ab/R35
18	10
20	13
22	11

Fig. 5E

Tube formation

Cont Ab	Cont Ab + CD5L	CD5L Ab/R35
65	95	39
51	106	25
50	75	27

Fig. 5F

Cell migration

Cont Ab	Cont Ab + CD5L	CD5L + R35 Ab
25	150	55
100	135	65
60	108	84
55	145	70
70	165	60
69	130	70
82	150	85
95	135	70
	120	60

Fig. 6C

Tumor weight(g)

Scramble + IgG	Scramble + B20	S76.T + IgG	S76.T + B20
1.09	1.18	0.15	0.2
1.27	0.53	0.85	0.06
2	0.24	0.14	0.25
0.93	0.94	0.8	0.03
0.5	0.45	0.33	0.04

Fig. 6D

Tumor nodules #

Scramble + IgG	Scramble + B20	S76.T + IgG	S76.T + B20
45	27	2	2
35	15	5	3
45	31	15	2
33	28	7	2
22	13	3	7

Fig. 6E

CD31

Scramble + IgG	Scramble + B20	S76.T + IgG	S76.T + B20
65	58	25	15
55	45	20	12
60	38	35	10
58	40	26	20

Fig. 6F

Ki67

Scramble + IgG	Scramble + B20	S76.T + IgG	S76.T + B20
85	65	65	25
88	70	71	40
75	82	70	32
70	60	55	35

Fig. 7A

CD5L expression

Responders	Non-responders
5	9.5
1.5	7
0	5.5
1	10
7	10
8	8.5
8	5
3	7
7	5
3	7
1	5
1	
1	
7	
10	
4	
4	
3	
1	
5	
4	
2	
0.5	
3	
4	

Fig. 7B

CD5L serum protein levels

Responders	Non-responders
2000	8000
3800	8000
3900	4000
4000	3900
1800	3950
1700	4000
1700	2200
1750	

Fig. 7D

Kaplan Meier curve of overall survival

Path_No	CD5L	HISTO	VITSTAT	SURVIVAL	group	os_status
SH01-5127	2	HG	Dead	408	high	1
SZ85-83	2	HG	Alive	303	high	0
SZ86-12295	3	BLT	Alive	281	high	0
SZ93-8314	3	HG	Alive	200	high	0
SH04-1647	2	HG	Dead	184	high	1
SZ97-10718	3	BLT	Alive	148	high	0
SH02-4270	2	HG	Dead	145	high	1
SZ98-5096	1	HG	Alive	142	low	0
SZ99-337	1	HG	Alive	135	low	0
SZ99-6908	2	BLT	Alive	128	high	0
SZ98-5458	2	HG	Alive	120	high	0
SZ00-4151	2	LG	Alive	119	high	0
SZ00-4196	2	BLT	Dead	119	high	1
SH00-14851	2	BLT	Alive	118	high	0
SH00-8781	2	HG	Alive	116	high	0
SZ93-8651	1		Dead	116	low	1
SZ95-5179	1	BLT	Alive	112	low	0
SZ01-84	2	HG	Alive	111	high	0
SZ93-5080	3	HG	Dead	106	high	1
SZ98-9147	1	BLT	Alive	103	low	0
SH01-14811	1	HG	Alive	103	low	0
SH02-2077	1	BLT	Alive	98	low	0
SH02-6737	2	HG	Alive	95	high	0
SZ99-9433	1	BLT	Dead	90	low	1
SH03-6935	1	HG	Alive	87	low	0
SZ93-10275	1	HG	Dead	80	low	1
SH03-16806	1	HG	Alive	79	low	0
SH03-20910	3		Alive	78	high	0
SH00-8804	2	HG	Dead	75	high	1
SH04-3767	2	HG	Alive	73	high	0
SZ97-1383	1	HG	Dead	73	low	1
SZ00-6132	1		Dead	71	low	1
SH03-16896	2	HG	Dead	70	high	1
SZ97-582	1	HG	Dead	69	low	1
SZ97-10074	1	HG	Dead	64	low	1
SZ99-2772	2	HG	Dead	62	high	1
SZ99-7739	1	BLT	Alive	61	low	0
SH01-5737	2	BLT	Dead	59	high	1
SH04-2723	3	HG	Dead	58	high	1
SZ99-1572	1	HG	Dead	57	low	1
SZ94-6823	2	HG	Dead	54	high	1
SZ00-6083	2	HG	Dead	53	high	1
SH02-14508	3	HG	Dead	52	high	1
SH04-249	3	HG	Dead	52	high	1
SZ99-9955	1	HG	Dead	50	low	1
SH03-2457	1	HG	Dead	46	low	1
SH97-10231	1	HG	Dead	45	low	1
SZ99-6778	2	HG	Dead	43	high	1
SZ89-479	1	HG	Dead	42	low	1
SZ87-7029	2	HG	Dead	42	high	1
SH02-9620	3	HG	Dead	41	high	1
SZ99-6095	1	HG	Dead	40	low	1
SH02-14153	2	HG	Dead	40	high	1
SZ98-7823	2		Dead	40	high	1
SH03-18808	2		Dead	40	high	1
SZ90-10518	2	HG	Dead	37	high	1
SZ89-8161	1	HG	Dead	35	low	1
SZ99-1980	2	HG	Dead	35	high	1
SZ99-9269	1	HG	Dead	33	low	1
SZ92-2527	3	HG	Dead	31	high	1
SH02-2385	3	HG	Dead	31	high	1
SH01-3787	2		Dead	29	high	1
SZ99-3009	2	HG	Dead	28	high	1
SZ99-9147	3	HG	Dead	28	high	1
SZ94-7961	3	HG	Dead	27	high	1
SH03-15629	3	HG	Dead	26	high	1
SH01-14758	1	HG	Dead	25	low	1
SH02-4619	2	HG	Dead	25	high	1
SH03-11959	1	HG	Dead	24	low	1
SZ89-14400	1	HG	Dead	23	low	1
SZ00-4249	1	HG	Dead	23	low	1
SZ99-9554	2	HG	Dead	23	high	1
SH04-1568	3	HG	Dead	22	high	1
SZ99-8077	1	HG	Dead	21	low	1
SZ94-5260	1	HG	Dead	21	low	1
SH01-14810	1	HG	Dead	21	low	1
SH03-18283	1	HG	Dead	21	low	1
SH04-2719	1		Dead	20	low	1
SZ98-9896	2	HG	Dead	20	high	1
SH02-13134	3	HG	Dead	20	high	1
SZ99-4745	3	HG	Dead	20	high	1
SZ93-10005	1	HG	Dead	19	low	1
SZ00-1758	1	HG	Dead	19	low	1
SH01-1548	2	HG	Dead	18	high	1
SZ97-6399	3		Dead	18	high	1
SZ00-2140	3	HG	Dead	18	high	1
SZ99-275	1	HG	Dead	17	low	1
SZ90-15399	3	HG	Dead	17	high	1
SZ94-9521	1	HG	Dead	16	low	1
SZ92-3034	3	HG	Dead	16	high	1
SZ95-359	2	HG	Dead	15	high	1
SH01-227	2	HG	Dead	15	high	1
SZ91-5035	2		Dead	15	high	1
SZ87-3548	2	HG	Dead	13	high	1
SH97-11103	3	HG	Dead	12	high	1
SZ91-14597	3	HG	Dead	11	high	1
SZ90-8015	2	HG	Dead	10	high	1
SZ00-2307	3	HG	Dead	9	high	1
SH01-8281	3	HG	Dead	9	high	1
SZ97-1668	2	HG	Dead	8	high	1
SZ91-7337	1	HG	Dead	7	low	1
SZ00-1757	2	HG	Dead	6	high	1
SZ99-5995	1	HG	Dead	2	low	1
SZ95-550	3	HG	Dead	1	high	1
SZ94-5510	3	HG	Dead	1	high	1
SZ93-10974	3	HG	Dead	1	high	1

Fig. S2

Tube formation

Scrambled Control	CD5L K/O cells	CD5L K/O cells + CD5L
41	28	32
45	15	38
26	28	40
33	22	33

Fig. S5A

mRNA expression

PPARG		HIF1A	
**Sensitive EC**	**Resistant EC**	**Sensitive EC**	**Resistant EC**
5.034663781	3.879800558	15.05285237	54.87663
4.961871	5.187056	26.90555	48.41929
3.421799	6.556556	42.98842	46.15758
			49.14191

Fig. S5B

mRNA expression

PPARG				CD5L			
**Normoxia**	**Hypoxia**	**Normoxia**	**Hypoxia**	**Normoxia**	**Hypoxia**	**Normoxia**	**Hypoxia**
1.066378	5.946482	1.087141	2.588485	1.324204	2.63623	1.201113	1.534317
1.179994	5.869752	0.942837	2.479953	1.018309	2.509022	0.832	1.436031
0.794711	6.256959	0.975613	2.452746	0.741593	2.570185		1.6467

Fig. S6A

RPPA assay

	AKTpS473	AKT pT308	MAPK PT202 Y204	P38 pT180 Y182	PKCα pS657	JNK pT183	Rictor pT1135	STAT3 pY705	AMPK pT172	PI3K p110α	PI3K p85
RF24 CD5L/RF24 EV #1	1.13528843	1.16573275	1.045680442	0.65577	1.10982	0.875424276	1.678717044	1.033136156	0.054221738	0.947899569	1.9548741
RF24 CD5L/RF24 EV #2	1.28287593	1.20070473	1.077050855	0.67544	1.14311	0.875424276	1.729078556	1.06413024	0.05584839	0.976336556	2.0135204
RF24 CD5L/RF24 EV #3	1.27663184	1.28230603	1.071299613	0.67183	1.13701	0.896872171	1.887717317	1.058447991	0.044391898	0.971123108	2.0027686

Fig. S6B

Tube formation

RF24	RF24 + CD5L
28	66
29	68
47	63

Fig. S6C

Cell migration

RF24 cells	RF24 + CD5L
130	100
10	120
90	110
60	115
68	115
80	100
83	140
85	158
72	150

Fig. S7B

Tube formation

Scrambled Control	PPARG K/O cells	PPARG K/O cells + CD5L
40	16	30
27	25	31
32	13	26

Fig. S8A

Tube formation

Control Ab	Control Ab + CD5L	R-35 Ab + CD5L
60	112	61
90	102	66
80	105	85
80	106	66

Fig. S8B

Tube formation

Control Ab	Control Ab +CD5L	R-35 Ab + CD5L
38	41	16
33	48	23
26	55	27
33	59	28

Fig. S8C

Capillary formation

Control Ab	Control Ab +CD5L	R-35 Ab + CD5L
73.15	200.09	60
70.11	183.81	73.09
69.21	152.11	47.41
80.25	150.55	60.57

Fig. S9B

WBC	
IgG	CD5L Ab
10.9	5.93
4.43	6.49
7.75	4.62
7.17	8.64

HGB	
IgG	CD5L Ab
14	14.2
12.2	11.2
14.7	13.9
13.3	14.2

ALT	
IgG	CD5L Ab
81	38
71	54
34	48
58	42

AST	
IgG	CD5L Ab
401	272
517	413
213	267
380	164

LDH	
IgG	CD5L Ab
1610	864
1494	1406
676	904
1289	2500

PLATELETS	
IgG	CD5L Ab
1344	1523
1100	1570
1157	1273
1400	1440

MONOS	
IgG	CD5L Ab
0.14	0.06
0.04	0.09
0.1	0.08
0.1	0.13

EOS	
IgG	CD5L Ab
0.37	0.24
0.27	0.44
0.2	0.25
0.24	0.17

Fig. S10B

Tumor weight (g)

**WT-R35**									
0.96	0.64	0.54	1.17	0.53	0.34	0.97	0.62	0.72	
**CD36loxfloxTie2Cre-R35**									
0.83	0.19	0.54	0.21	0.5	1.5	0.37	0.2		
**CD36loxfloxTie2Cre-IgG**									
0.74	0.51	1.54	1	0.63	0.72	0.18	0.54		

Tumor Nodule #

**WT-R35**									
25	15	27	32	12	25	20	22	29	
**CD36loxfloxTie2Cre-R35**									
20	4	18	2	14	31	4	11		
**CD36loxfloxTie2Cre-IgG**									
18	20	42	29	15	25	2	18		

Ascites (ml)

**WT-R35**									
3.5	5	2	9.4	4.8	3.5	4.5	3.2	0	
**CD36loxfloxTie2Cre-R35**									
2	2.2	3.2	9	1.2	6	3	2.8		
**CD36loxfloxTie2Cre-IgG**									
4.8	5.2	7	5.6	4.7	1.5	0	1		

Fig. S10C

Mouse weight (g)

**WT-R35**								
26.77	25.88	23.2	32.14	30.13	25.06	28.3	24	25.19
**CD36loxfloxTie2Cre-R35**								
20.3	18.28	24.53	34.65	18.38	28.76	22.41	22.78	
**CD36loxfloxTie2Cre-IgG**								
27.7	31.21	31.7	31.05	29.29	26.81	27.93	28.86	

Fig. S10F

DAY	WT-R35										
0		0	0	0	0	0	0	0	0	0	0
7		0	0	0	0	2.3	0	0	0	0	1.1
18		5.2	1	1	1	11	2.3	0	0	0	1.2
35		5.2	56.3	212	89	108	120	125	89	111	165

DAY	CD36loxfloxTie2Cre-R35									
0	0	0	0	0	0	0	0	0		
7	2.5	2.6	2.8	3.5	5.6	0	0.8	0		
18	5.8	20.6	45.5	5.2	11.4	12.6	23	1		
35	200	180	175	205	355	56	25	203		

DAY	CD36loxfloxTie2Cre-IgG							
0	0	0	0	0	0	0	0	0
7	0	5.3	0	0	6.28	0	0	2.2
18	4.6	1	1	2.6	11.2	23.2	8.5	19.8
35	202	89	56	98	135	125	255	200

Fig. S12B

30 minutes

	control siRNA						CD36 siRNA					
untreated	27891	29596	31168	27256	24172	30113	31061	28377	29610	32586	35414	33662
rCD5L	54528	52561	54725	52660	53746	52264	25089	18383	21268	23382	26938	26562
R35	19790	15481	19447	17666	10990	18835	17097	16655	16284	17590	16507	16228

60 minutes

	control siRNA						CD36 siRNA					
untreated	51119	53875	55643	52205	52944	53619	50925	52066	49578	51897	54480	50288
rCD5L	112341	113128	118529	121289	109832	109332	51279	46325	49543	52710	46328	48035
R35	49096	48206	52738	50984	45074	52610	51184	50872	51219	51057	51225	49743

Fig. S14B

Raw data, normalized fluorescence

Titrant Conc. / nM	Tech 1	Tech 2
0,27	771,238	771,951
0,55	770,912	771,339
1,1	771,715	771,026
2,2	770,067	770,178
4,39	771,366	771,398
8,79	770,115	769,538
17,58	771,848	770,724
35,16	771,364	772,526
70,31	771,209	769,54
140,63	772,154	772,386
281,25	773,91	774,135
562,5	775,252	775,361
1125	776,175	778,322
2250	777,638	778,081
4500	778,488	778,778
9000	779,315	778,607

Fig. S17A

Titrant Conc. / nM	Tech 1	Tech 2
0,015	9,867,356	9,732,279
0,03	10,058,943	10,236,875
0,061	9,902,555	9,782,347
0,122	10,454,075	10,441,252
0,244	10,237,094	10,191,662
0,488	10,734,435	10,579,586
0,977	11,122,175	10,955,834
1,953	11,277,214	11,241,025
3,906	11,434,924	11,302,659
7,812	12,122,485	12,115,199
15,625	12,834,644	12,732,886
31,25	12,536,791	12,599,306
62,5	13,092,444	13053,41
125	12,548,939	12,539,506
250	12,557,033	12,533,906
500	13,038,658	13,094,747

Fig. S17C

Titrant Conc. / nM	Tech 1	Tech 2
0,0305	892,8	892,49
0,061	892,607	892,148
0,122	892,788	892,337
0,244	892,706	892,529
0,488	892,127	892,066
0,977	892,906	892,563
1,953	893,012	893,677
3,906	893,498	893,088
7,812	894,062	894,186
15,625	894,628	895,172
31,25	895,02	894,654
62,5	895,948	895,181
125	896,467	895,71
250	896,377	895,898
500	895,778	895,907
1,000	896,713	896,349

Fig. S18B

Tube formation

Scramble	S76.T
85	50
80	45
70	55

Fig. S18C

Cell migration

Scramble	S76.T
130	25
125	28
133	30

Fig. S18D

Proliferation assay

Scramble + IgG	Scramble + Bevacizumab	S76.T + IgG	S76.T + Bevacizumab
102.57	89.39	57.3	51.57
95.7	92.26	54.44	42.4
105.44	86.53	63.03	42.4
96.27	80.22	51.57	44.69

Table 1.Kd Binding Values for S5, S47, S63 and S72 Obtained by MTS.

Sequence Name	Kd vs CDL5	Kd vs VEGF
S5	555.8±148.9 nM	Weak binding, not fit with the law of mass action
S47	1552.4±309.4 nM	103.4±45.4 nM
S63	No binding between 500 pM to 50 mM	Weak binding, not fit with the law of mass action
S72	Weak binding, not fit with the law of mass action	135.2±61.6 nM

Table 2. Kd Binding Values for S11, S23 and S76 Obtained by MTS

Sequence Name	Kd vs CDL5	Kd vs VEGF
S11	59.2±22.14 nM	Weak binding, not fit with the law of mass action
S23	92.1±40.1 nM	Weak binding, not fit with the law of mass action
S76	2.2±0.7 nM	Weak binding, not fit with the law of mass action

Table 3.Quantitative-PCR primer lists.

No.	Primers	Sequence (5'-3')
1	Human CD5L F	5'- CTGCTTGTTCTCCTGAGCCC -3'
2	Human CD5L R	5'- TCAAAGGGTCAGGGTTGAGC -3'
3	Human PPARG-F	5'- GCCCTTTGGTGACTTTATGGA -3'
4	Human PPARG-R	5'- GCAGCAGGTTGTCTTGGATG -3'
5	Human CD36 F	5'- GAGAACTGTTATGGGGCTAT -3'
6	Human CD36 R	5'- TTCAACTGGAGAGGCAAAGG -3'
7	Tie2 Cre transgene F	5'-CGCATAACCAGTGAAACAGCATTGC-3'
8	Tie2 Cre transgene R	5'-CCCTGTGCTCAGACAGAAATGAGA-3'
